# First dose ChAdOx1 and BNT162b2 COVID-19 vaccinations and cerebral venous sinus thrombosis: A pooled self-controlled case series study of 11.6 million individuals in England, Scotland, and Wales

**DOI:** 10.1371/journal.pmed.1003927

**Published:** 2022-02-22

**Authors:** Steven Kerr, Mark Joy, Fatemeh Torabi, Stuart Bedston, Ashley Akbari, Utkarsh Agrawal, Jillian Beggs, Declan Bradley, Antony Chuter, Annemarie B. Docherty, David Ford, Richard Hobbs, Srinivasa Vittal Katikireddi, Emily Lowthian, Simon de Lusignan, Ronan Lyons, James Marple, Colin McCowan, Dylan McGagh, Jim McMenamin, Emily Moore, Josephine-L. K. Murray, Rhiannon K. Owen, Jiafeng Pan, Lewis Ritchie, Syed Ahmar Shah, Ting Shi, Sarah Stock, Ruby S. M. Tsang, Eleftheria Vasileiou, Mark Woolhouse, Colin R. Simpson, Chris Robertson, Aziz Sheikh

**Affiliations:** 1 Usher Institute, The University of Edinburgh, Edinburgh, United Kingdom; 2 Nuffield Department of Primary Care Health Sciences, University of Oxford, Oxford, United Kingdom; 3 Population Data Science, Swansea University Medical School, Swansea, United Kingdom; 4 School of Medicine, University of St. Andrews, St Andrews, United Kingdom; 5 BREATHE–The Health Data Research Hub for Respiratory Health, University of Edinburgh, Edinburgh, United Kingdom; 6 Queen’s University Belfast, Belfast, United Kingdom; 7 Public Health Agency, Belfast, United Kingdom; 8 MRC/CSO Social & Public Health Sciences Unit, Glasgow, United Kingdom; 9 Royal Infirmary of Edinburgh, NHS Lothian and Anaesthesia, Critical Care and Pain Medicine, The University of Edinburgh, Edinburgh, United Kingdom; 10 Public Health Scotland, Glasgow, United Kingdom; 11 Department of Mathematics and Statistics, University of Strathclyde, Glasgow, United Kingdom; 12 Academic Primary Care, University of Aberdeen School of Medicine and Dentistry, Aberdeen, United Kingdom; 13 School of Health, Wellington Faculty of Health, Victoria University of Wellington, New Zealand; Leiden University Medical Center, NETHERLANDS

## Abstract

**Background:**

Several countries restricted the administration of ChAdOx1 to older age groups in 2021 over safety concerns following case reports and observed versus expected analyses suggesting a possible association with cerebral venous sinus thrombosis (CVST). Large datasets are required to precisely estimate the association between Coronavirus Disease 2019 (COVID-19) vaccination and CVST due to the extreme rarity of this event. We aimed to accomplish this by combining national data from England, Scotland, and Wales.

**Methods and findings:**

We created data platforms consisting of linked primary care, secondary care, mortality, and virological testing data in each of England, Scotland, and Wales, with a combined cohort of 11,637,157 people and 6,808,293 person years of follow-up. The cohort start date was December 8, 2020, and the end date was June 30, 2021. The outcome measure we examined was incident CVST events recorded in either primary or secondary care records. We carried out a self-controlled case series (SCCS) analysis of this outcome following first dose vaccination with ChAdOx1 and BNT162b2. The observation period consisted of an initial 90-day reference period, followed by a 2-week prerisk period directly prior to vaccination, and a 4-week risk period following vaccination. Counts of CVST cases from each country were tallied, then expanded into a full dataset with 1 row for each individual and observation time period. There was a combined total of 201 incident CVST events in the cohorts (29.5 per million person years). There were 81 CVST events in the observation period among those who a received first dose of ChAdOx1 (approximately 16.34 per million doses) and 40 for those who received a first dose of BNT162b2 (approximately 12.60 per million doses). We fitted conditional Poisson models to estimate incidence rate ratios (IRRs). Vaccination with ChAdOx1 was associated with an elevated risk of incident CVST events in the 28 days following vaccination, IRR = 1.93 (95% confidence interval (CI) 1.20 to 3.11). We did not find an association between BNT162b2 and CVST in the 28 days following vaccination, IRR = 0.78 (95% CI 0.34 to 1.77). Our study had some limitations. The SCCS study design implicitly controls for variables that are constant over the observation period, but also assumes that outcome events are independent of exposure. This assumption may not be satisfied in the case of CVST, firstly because it is a serious adverse event, and secondly because the vaccination programme in the United Kingdom prioritised the clinically extremely vulnerable and those with underlying health conditions, which may have caused a selection effect for individuals more prone to CVST. Although we pooled data from several large datasets, there was still a low number of events, which may have caused imprecision in our estimates.

**Conclusions:**

In this study, we observed a small elevated risk of CVST events following vaccination with ChAdOx1, but not BNT162b2. Our analysis pooled information from large datasets from England, Scotland, and Wales. This evidence may be useful in risk–benefit analyses of vaccine policies and in providing quantification of risks associated with vaccination to the general public.

## Abstract 2

In a pooled self-controlled case series study, Steven Kerr and colleagues assess the association between first dose ChAdOx1 and BNT162b2 COVID-19 vaccination with cerebral venous sinus thrombosis in England, Scotland and Wales.

## Author summary

**Why was this study done?:**

- There have been indications of a possible association between Coronavirus Disease 2019 (COVID-19) vaccines—in particular, Oxford/AstraZeneca (ChAdOx1)—and cerebral venous sinus thrombosis (CVST), which is a rare type of blood clot in the brain.
- Further evidence on this topic is required in order to inform vaccine policy deliberations.

**What did the researchers do and find?:**

- We estimated the rate of CVST events in the 4 weeks following first dose vaccination compared with a 90-day period prior to vaccination.
- The rate of CVST events in the 4 weeks following vaccination with Oxford/AstraZeneca was approximately twice as high compared to the baseline rate, implying an additional 0.25 events on average in this period per million people vaccinated.
- We did not find an association between Pfizer-BioNTech (BNT162b2) and CVST.

**What do these findings mean?:**

- We found evidence of a slight increased risk of CVST following first dose vaccination with ChAdOx1, but not BNT162b2.
- Public health authorities may wish to take this evidence into account when communicating the benefits and risks associated with COVID-19 vaccines to the general public.
- Our study had some limitations. Although the cohort was large, CVST is an extremely rare event, and this may have caused some imprecision in our estimates. In addition, there is a possibility that our model assumptions were not satisfied.

## Introduction

There have been concerns over possible associations between some Coronavirus Disease 2019 (COVID-19) vaccines and hematological and vascular adverse events, including, in particular, cerebral venous sinus thrombosis (CVST) following ChAdOx1 nCoV-19 (Oxford/AstraZeneca; henceforth ChAdOx1). A number of case reports and case series of CVST following adenovirus vector COVID-19 vaccination have been published [1–5]. A potential link between ChAdOx1 was initially noted by the European Medicines Agency (EMA) in a safety update on March 29, 2021 [6]. On April 8, 2021, the EMA issued an analysis of pharmacovigilance data covering the European Economic Area that found a safety signal for CVST following ChAdOx1 vaccination, risk ratio = 7.73 (95% confidence interval (CI) 5.35 to 10.80) [7]. An observed versus expected analysis using data from the Mayo Clinic Health System in the United States found a relative risk of 1.50 (95% CI 0.28 to 7.10) of CVST in a combined analysis of COVID-19 vaccines [8]. An observed versus expected analysis in Denmark and Norway found a standardised morbidity ratio of 20.25 (95% CI 8.14 to 41.73) for cerebral venous thrombosis following ChAdOx1 vaccination [9]. A self-controlled case series (SCCS) using the QResearch database in England found incidence rate ratio (IRR) in the 8 to 28 days following vaccination of 2.37 (95% CI 1.34 to 4.21) for ChAdOx1 and 1.93 (95% CI 0.87 to 4.28) for BNT162b2 [10]. Several countries suspended ChAdOx1 or restricted its use to older age groups due to these and other safety concerns [11]. The Joint Committee on Vaccination and Immunisation (JCVI), an independent UK-wide body that advises the government on vaccine approval, has recommended that adults under the age of 40 should be offered an alternative to the ChAdOx1 vaccine, if available [12,13].

At the time of writing, 3 vaccines are being administered in the UK: ChAdOx1, BNT162b2 (Pfizer-BioNTech), and mRNA-1273 (Moderna). They have all shown high levels of efficacy in Phase II and III clinical trials [14–16]. ChAdOx1 and BNT162b2 have also shown high levels of “real-world effectiveness” against COVID-19 hospitalisation and death [17,18]. Vaccine rollout in the UK started with BNT162b2 on December 8, 2020, followed by ChAdOx1 on January 4, 2021 and mRNA-1273 on April 7, 2021. Guidance from the JCVI included a list of priority groups with the elderly, frontline social and health care workers, and the clinically extremely vulnerable at the highest levels of priority (S2 File). Relatively few people in our cohort were vaccinated with mRNA-1273, so this study focused on BNT162b2 and ChAdOx1 only.

The aim of this study was to investigate possible associations between COVID-19 vaccines and CVST. We carried out a SCCS study of CVST events following first dose vaccination with ChAdOx1 and BNT162b2. The data platform used in this study consisted of linked primary care, secondary care, mortality and virological testing data stored in secure trusted research environments (TREs) in each of England, Scotland, and Wales. CVST is an extremely rare event, with an estimated incidence of 3 to 4 per million person years in adults [19]. In our previous analysis exploring COVID-19 vaccine associations with thrombocytopenic, thromboembolic, and hemorrhagic events using Scottish national data, there were insufficient CVST events to undertake a statistical analysis [20]. We were not able to reliably estimate the association between COVID-19 vaccines and CVST with any individual country-specific dataset due to the low number of events. In order to address this, we pooled incident CVST cases from each of the datasets and carried out a SCCS analysis to estimate IRRs for CVST in those who received a first dose of ChAdOx1 or BNT162b2 vaccines.

## Methods

### Study design and population

**Fig 1:**
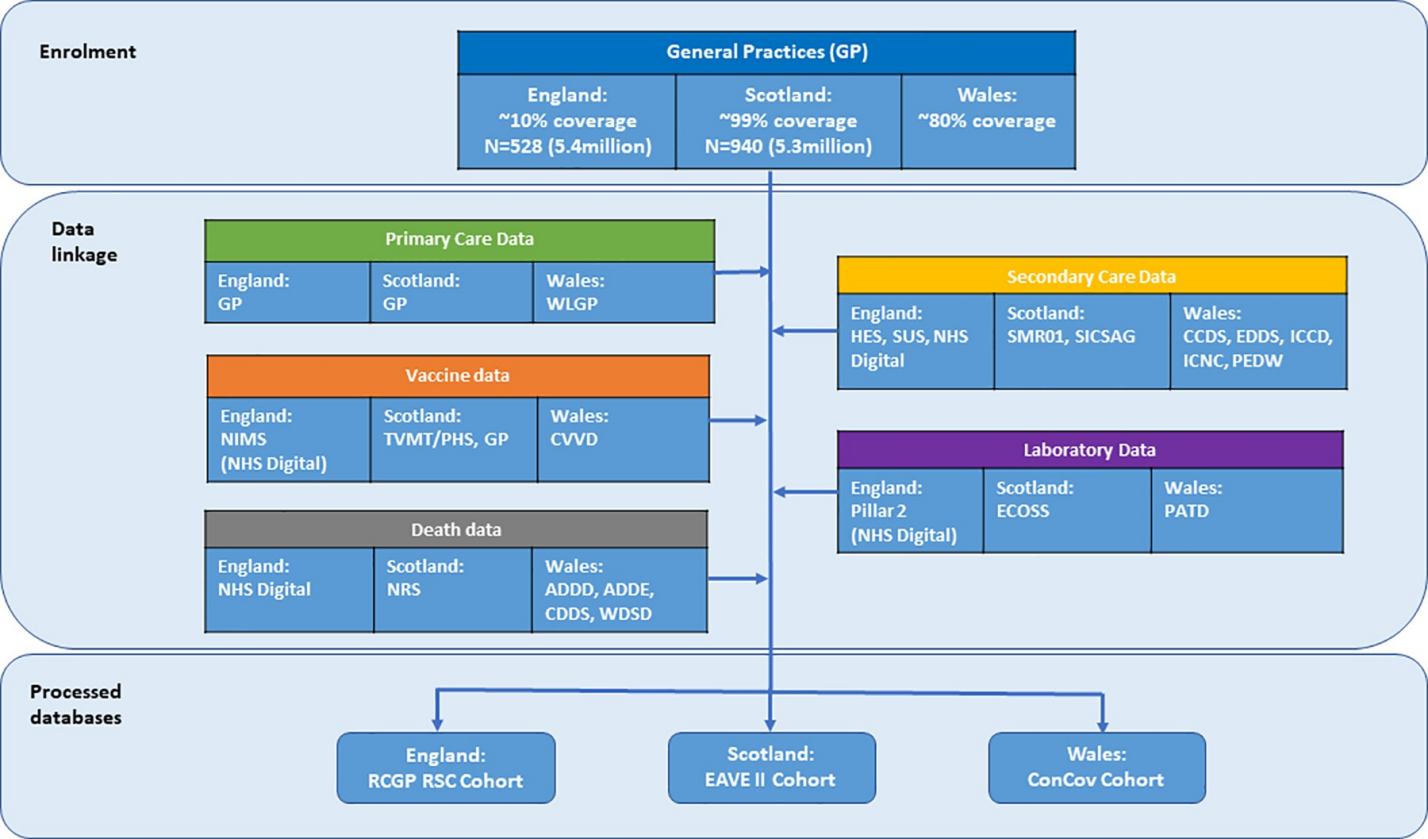
Data linkage diagram. ADDD, Annual District Death Daily; ADDE, Annual District Death Extract; CCDS, Critical Care Dataset; CDDS, Consolidated Death Data Source; ConCov, Controlling COVID-19 through enhanced population surveilance and intervention; CVVD, COVID-19 Vaccine Data; EAVE, Early Assessment of Vaccine and antiviral Effectiveness; ECOSS, Electronic Communication of Surveillance in Scotland; EDDS, Emergency Department Dataset; GP, general practices; HES, Hospital Episode Statistics; ICCD, ICNARC – Intensive Care National Audit & Research Centre (COVID only admissions); ICNC, Intensive Care National Audit & Research Centre; NHS, National Health Service; NIMS, National Immunisation Management System; NRS, National Records of Scotland; ONS, Office for National Statistics; PATD, Pathology data COVID-19 Daily; PEDW, Patient Episode Database for Wales; PHS, Publish Health Scotland; RCGP RSC, Oxford-Royal College of General Practitioners Research and Surveillance Centre; SICSAG, Scottish Intensive Care Society Audit Group; SMR01, Scottish Morbidity Records 01; SUS, Secondary Users Service; TVMT, Turas Vaccine Management Tool; WDSD, Welsh Demographic Service Dataset; WLGP, Welsh Longitudinal General Practice Dataset.

We followed a prespecified statistical analysis plan (S3 File). The datasets consisted of linked primary care, secondary care, mortality, and virological testing data stored in secure TREs in each of England, Scotland, and Wales (Fig 1). These data were deterministically linked using unique patient identifiers—NHS number in England and Community Health Index (CHI) number in Scotland. In Wales, a combination of deterministic linkage based on NHS number and probabilistic linkage based personal identifiers was used. Anyone under the age of 16 at the date of event was excluded. A case was defined as anyone in our cohorts with a CVST event following the start of the COVID-19 vaccination programme in the UK. An incident case was defined as the first such event in the cohort time period. The cohort start date was December 8, 2020, and the end date was June 30, 2021.

In accordance with our statistical analysis plan, we initially sought to carry out a meta-analysis of estimates from each country. However, there were too few events in each country for this to be feasible. As a result, we undertook a pooled analyses of aggregate count data from each country. We sought to carry out both a SCCS study and a case–control study using pooled data. However, the case–control study would have required sharing of individual-level information, which was not permitted under the data governance rules implemented by each country’s TREs. Therefore, we focused on the pooled SCCS study.

**Fig 2:**
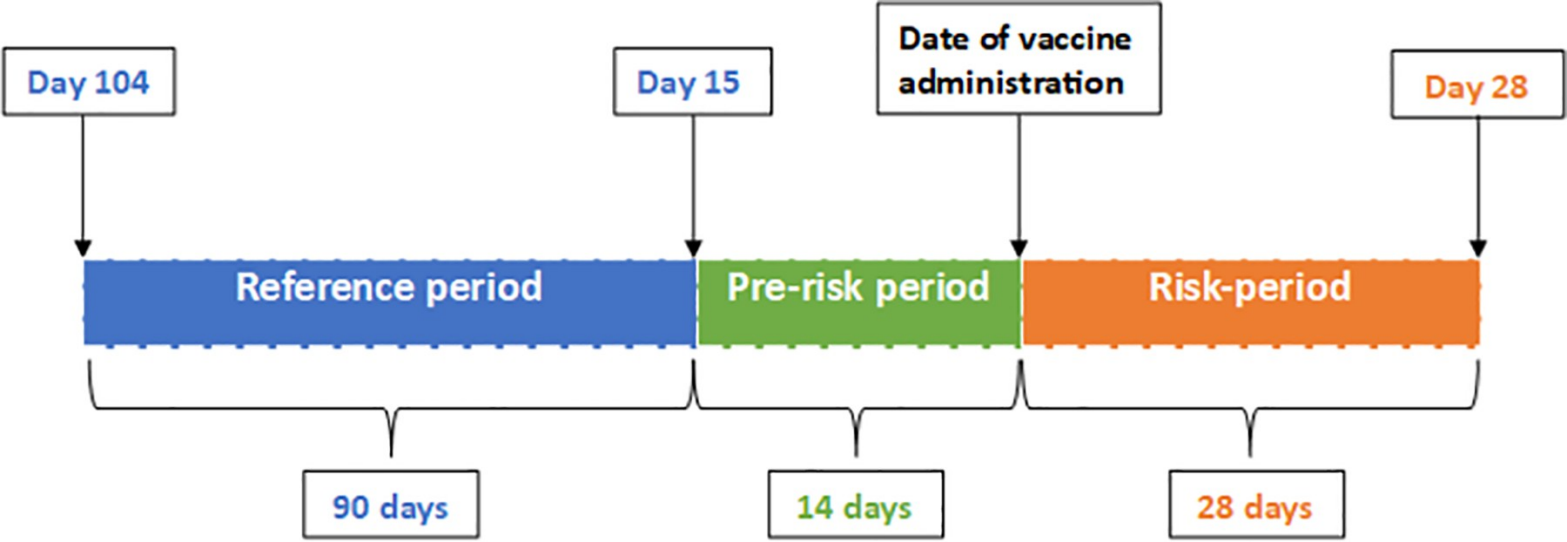
SCCS study design. SCCS, self-controlled case series.

**Fig 3:**
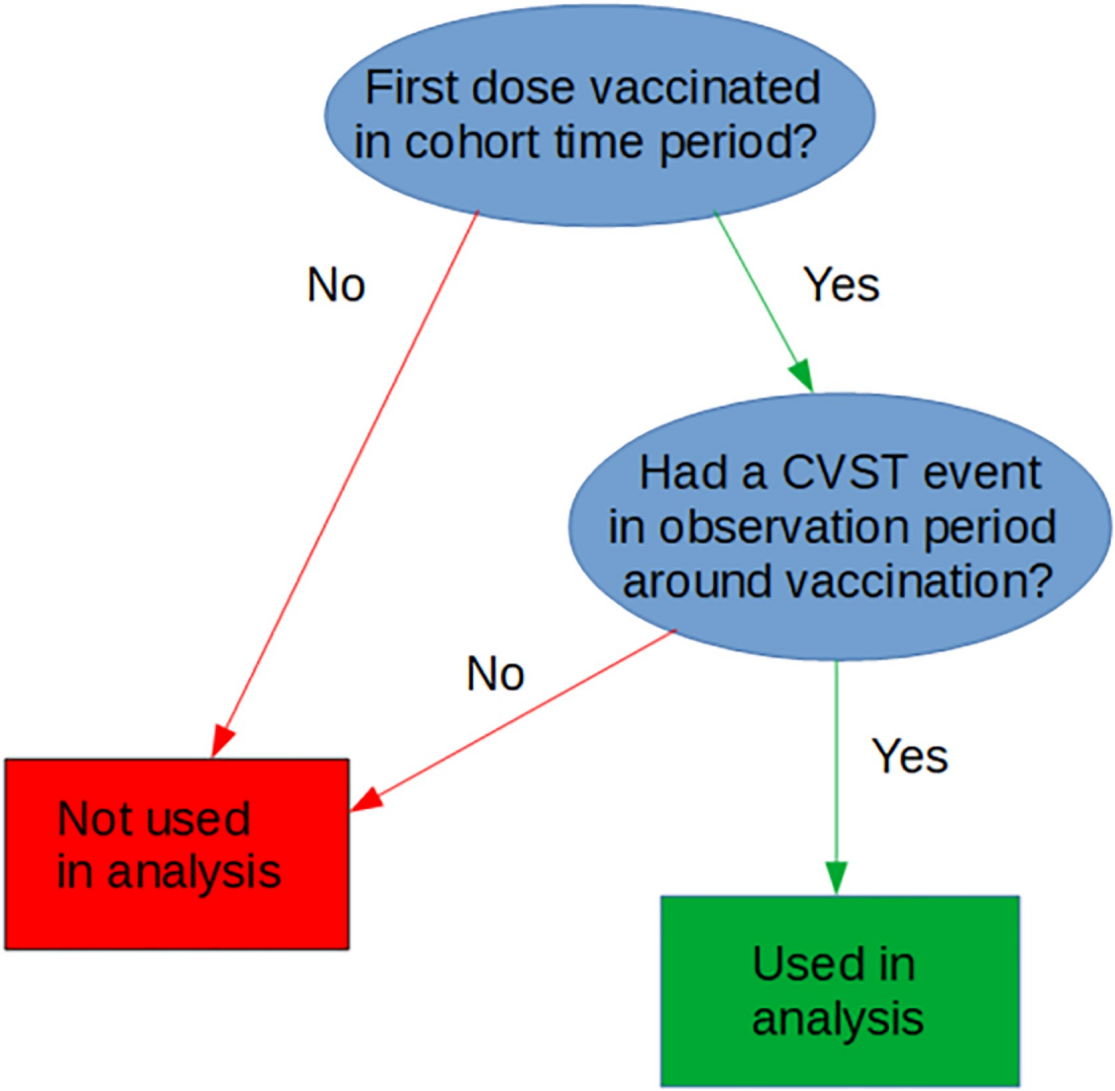
Selection procedure. CVST, cerebral venous sinus thrombosis.

The observation period for the SCCS started 104 days before first dose vaccination and ended 28 days after first dose vaccination (Fig 2). The reference period was defined as the first 90 days of the observation period. The prerisk period was defined as the 14-day period prior to first dose vaccination. The risk period was defined as 0 to 28 days post–first dose vaccination. No individual was censored because we examined incident cases only. We pooled data across the 3 nations on incident cases during the observation period around first dose vaccination with ChAdOx1 or BNT162b2 (Fig 3). Each stratum consisted of an incident case during the reference, prerisk, and risk periods.

### Exposure

We defined an individual as exposed from the day they were administered their first dose of either the BNT162b2 or ChAdOx1 vaccines.

### Outcomes

The outcome of interest was incident CVST cases in the observation period. SNOMED codes were used to identify CVST events recorded in primary care electronic health records in England, and Read Codes (Version 2) were used in Scotland and Wales (S4 File). International Classification of Diseases-10th Revision (ICD-10) codes were used to identify CVST events in hospital admission records (S4 File). SNOMED and Read Codes have hierarchies that enable thromboembolic events to be grouped by their location. These enable recording of the diagnosis of an event of interest—for example, a thromboembolic event could be recorded as intracranial, with further subdivisions into CVST, intracranial thrombophlebitis, and thrombosis of cerebral veins, within the SNOMED hierarchy. The code lists used in this study were drawn up by the Early Assessment of Vaccine and antiviral Effectiveness (EAVE) II clinical team and validated by experts in neurology and hematology.

### Statistical analysis

The data governance procedure of the TREs in each country did not allow for individual level data to be shared. However, we obtained permissions to confidentially share counts of individuals stratified by categories, on the understanding that these would be combined in a pooled count. For a SCCS study of the rate ratios (RRs) of incident events in the observation time periods that does not include any covariates, the only information that is required to estimate the conditional Poisson model is event counts in the reference, prerisk, and risk periods. Analysts in each country had full access to their country’s data. Counts of incident cases in the reference, prerisk, and risk periods stratified by vaccine type received were collated in each nation. These were then gathered in the Scottish TRE and expanded into a full dataset with 1 row per individual and observation time period. Synthetic IDs were generated in order to distinguish distinct incident cases in the count data from each other and to label the strata in the model. The process of pooling the data is explained in more detail in the Supporting information (S5 File). IRRs and their 95% CIs were estimated using a conditional Poisson model, with cases during the reference, prerisk, and risk periods as the strata and an offset for the length of each time period. The reference period was taken as the baseline period for calculating IRRs. Conditional Poisson models have a likelihood function that is identical to the corresponding conditional logistic model [21]. Furthermore, incident events can occur at most once. Thus, we used the clogit function for conditional logistic regression from the survival package in R to fit the model. Following a suggestion from one of the reviewers, we undertook 2 post hoc sensitivity analyses: The first excluded any cases who died within 90 days of their event in order to explore the SCCS assumption of event-dependent exposure, and the second focused only on CVST cases identified in secondary care records.

### Ethics and permission

In England, approvals were obtained from the Health Research Authority, London Central (reference number 21/HRA/2786). In Scotland, data approvals were obtained from the National Research Ethics Service Committee, Southeast Scotland 02 (reference number: 12/SS/0201), and Public Benefit and Privacy Panel for Health and Social Care (reference number: 1920–0279). In Wales, approval was provided by SAIL independent Information Governance Review Panel (IGRP) (Project 0911).

### Reporting

This study is reported in accordance with the REporting of studies Conducted using Observational Routinely-collected Data (RECORD) guidelines (S1 File) [22,23].

## Results

The cohorts started on December 8, 2020 and ended on June 30, 2021. Tables 1–3 show the marginal distributions of a number of characteristics in each country’s cohort. Clinical risk groups in these tables were derived from the QCovid algorithm [24]. Among the approximately 4.95 million people vaccinated with ChAdOx1, there were 45 incident cases of CVST during the 90-day reference period and 27 incident cases of CVST during the 28-day postvaccine risk period. We found an IRR of 1.93 (95% CI 1.20 to 3.11) in the risk period following first dose vaccination with ChAdOx1 and an IRR of 0.78 (95% CI 0.34 to 1.77) in the risk period following first dose vaccination with BNT162b2 (Table 4). Assuming a baseline incidence of 3 to 4 cases of CVST per million people per year outside of the risk period, our estimates imply an absolute risk of 0.44 to 0.59 incident CVST cases per million people in the 4-week risk period following vaccination with ChAdOx1.

**Table 1:** Cohort summary statistics, England.

Characteristic	Level	Unvaccinated	One-dose ChAdOx1	One-dose BNT162b2
Total		1,322,305	2,247,155	1,629,360
Person years of follow-up		745,303	1,269,229	927,224
Incident CVST cases		27	49	30
Deaths		16,369 (1.2%)	11,327 (0.5%)	8,066 (0.5%)
Sex	Female	649,546 (49.1%)	1,176,988 (52.4%)	924,283 (56.7%)
	Male	672,759 (50.9%)	1,070,167 (47.6%)	705,077 (43.3%)
Age (years)	Mean (SD)	38.58 (15.54)	56.49 (14.77)	52.56 (21.67)
Age group (years)	18 to 64	1,222,023 (92.4%)	1,576,834 (70.2%)	1,067,807 (65.5%)
	65 to 79	69,637 (5.3%)	558,596 (24.9%)	318,241 (19.5%)
	80+	30,645 (2.3%)	111,725 (5.0%)	243,312 (14.9%)
Deprivation status^†^	1 (high)	303,447 (22.9%)	323,925 (14.4%)	232,356 (14.3%)
	2	295,143 (22.3%)	381,384 (17.0%)	290,696 (17.8%)
	3	253,275 (19.2%)	451,355 (20.1%)	333,697 (20.5%)
	4	239,858 (18.1%)	512,133 (22.8%)	368,663 (22.6%)
	5 (low)	229,464 (17.4%)	576,815 (25.7%)	402,905 (24.7%)
	Unknown	1,118 (0.1%)	1,543 (0.1%)	1,043 (0.1%)
Urban/rural index	Rural town and fringe	324,138 (14.4%)	221,355 (13.6%)	106,384 (8.0%)
	Rural town and fringe in a sparse setting	32,629 (1.5%)	21,147 (1.3%)	8,133 (0.6%)
	Rural village and dispersed	51,315 (2.3%)	34,761 (2.1%)	19,813 (1.5%)
	Rural village and dispersed in a sparse setting	8,122 (0.4%)	5,633 (0.3%)	4,225 (0.3%)
	Urban city and town	1,116,335 (49.7%)	783,133 (48.1%)	591,421 (44.7%)
	Urban city and town in a sparse setting	10,593 (0.5%)	6,858 (0.4%)	3,384 (0.3%)
	Urban major conurbation	614,524 (27.3%)	494,346 (30.3%)	549,592 (41.6%)
	Urban minor conurbation	81,632 (3.6%)	56,723 (3.5%)	35,933 (2.7%)
	Unknown	7,867 (0.4%)	5,404 (0.3%)	3,420 (0.3%)
Number of risk groups^‡^	0	908,219 (68.7%)	1,165,109 (51.8%)	807,851 (49.6%)
	1	304,222 (23.0%)	661,515 (29.4%)	475,206 (29.2%)
	2	76,147 (5.8%)	255,007 (11.3%)	199,650 (12.3%)
	3	19,985 (1.5%)	96,980 (4.3%)	83,191 (5.1%)
	4	7,719 (0.6%)	40,388 (1.8%)	36,949 (2.3%)
	5+	6,013 (0.5%)	28,156 (1.3%)	26,513 (1.6%)
Number of previous tests^§^	0	1,147,503 (86.8%)	1,889,412 (84.1%)	1,350,615 (82.9%)
	1	119,612 (9.0%)	235.388 (10.5%)	183.933 (11.3%)
	2	34,089 (2.6%)	66,502 (3.0%)	53,012 (3.3%)
	3	10,610 (0.8%)	23,322 (1.0%)	17,770 (1.1%)
	4 to 9	10,442 (0.8%)	32,427 (1.4%)	23,953 (1.5%)
	10+	49 (0.0%)	104 (0.0%)	77 (0.0%)
Average household age	Mean (SD)	32.6 (17.5)	47.07 (21.61)	45.91 (24.88)
Number of people in household	1	338,168 (25.6%)	556,975 (24.8%)	429,940 (26.4%)
	2	261,989 (19.8%)	698,293 (31.1%)	499,565 (30.7%)
	3 to 5	551,966 (41.7%)	859,932 (38.3%)	602,873 (37.0%)
	6 to 10	129,832 (9.8%)	100,041 (4.5%)	75,591 (4.6%)
	11 to 30	18,660 (1.4%)	17,587 (0.8%)	9,650 (0.6%)
	31 to 100	6,485 (0.5%)	12,837 (0.6%)	6,950 (0.4%)
	101+	15,205 (1.1%)	1,490 (0.1%)	4,791 (0.3%)
BMI	Underweight	115,851 (8.8%)	46,480 (2.1%)	75,842 (4.7%)
	Normal weight	626,235 (47.4%)	757,023 (33.7%)	636,389 (39.1%)
	Overweight	359,299 (27.2%)	809,120 (36.0%)	527,780 (32.4%)
	Obese	220,920 (16.7%)	634,532 (28.2%)	389,349 (23.9%)
Smoking status	Ex-smoker	308,038 (23.3%)	657,234 (29.2%)	438,683 (26.9%)
	Nonsmoker	241,142 (18.2%)	1,241,281 (55.2%)	952,839 (58.5%)
	Smoker	714,755 (54.1%)	338,007 (15.0%)	209,358 (12.8%)
	Unknown	58,370 (4.4%)	10,633 (0.5%)	28,480 (1.7%)
Atrial fibrillation		11,713 (0.9%)	68,860 (3.1%)	74,990 (4.6%)
Asthma		180,744 (13.7%)	342,874 (15.3%)	264,060 (16.2%)
Blood cancer		4,066 (0.3%)	20,228 (0.9%)	18,117 (1.1%)
Heart failure		8,217 (0.6%)	40,231 (1.8%)	39,508 (2.4%)
Cerebral palsy		724 (0.1%)	3,326 (0.1%)	1,442 (0.1%)
Coronary heart disease		17,102 (1.3%)	100,989 (4.5%)	99,116 (6.1%)
Cirrhosis		1,678 (0.1%)	6,829 (0.3%)	4,598 (0.3%)
Congenital heart disease		4,399 (0.3%)	15,787 (0.7%)	10,637 (0.7%)
COPD		12,266 (0.9%)	69,891 (3.1%)	57,883 (3.6%)
Dementia		7,468 (0.6%)	29,271 (1.3%)	23,567 (1.4%)
Diabetes type 1		16,158 (0.7%)	16,158 (0.7%)	11,773 (0.7%)
Diabetes type 2		34,079 (2.6%)	185,353 (8.2%)	153,568 (9.4%)
Epilepsy		22,824 (1.7%)	52,820 (2.4%)	33,655 (2.1%)
Fracture		35,154 (2.7%)	89,439 (4.0%)	73,717 (4.5%)
Neurological disorder		1,964 (0.1%)	9,885 (0.4%)	6,276 (0.4%)
Parkinson disease		1,483 (0.1%)	8,338 (0.4%)	6,571 (0.4%)
Pulmonary hypertension		1,751 (0.1%)	8,106 (0.4%)	8,079 (0.5%)
Pulmonary rare		2,635 (0.2%)	16,128 (0.7%)	14,666 (0.9%)
Peripheral vascular disease		3,856 (0.3%)	20,518 (0.9%)	18,554 (1.1%)
Rheumatoid arthritis or SLE		7,909 (0.6%)	39,102 (1.7%)	27,449 (1.7%)
Respiratory cancer		1,891 (0.1%)	7,224 (0.3%)	5,814 (0.4%)
Severe mental illness		157,557 (11.9%)	387,974 (17.3%)	236,108 (14.5%)
Sickle cell disease		794 (0.1%)	1,731 (0.1%)	1,236 (0.1%)
Stroke/TIA		12,140 (0.9%)	64,068 (2.9%)	60,588 (3.7%)
Thrombosis or pulmonary embolus		5,219 (0.4%)	23,610 (1.1%)	18,864 (1.2%)
Care housing category	Care home	5,168 (0.4%)	21,534 (1.0%)	10,419 (0.6%)
	Homeless	5,026 (0.4%)	3,585 (0.2%)	1,563 (0.1%)
Learning disability or Down syndrome	Learning disability	15,620 (1.2%)	39,679 (1.8%)	23,426 (1.4%)
	Down syndrome	271 (0.0%)	1,857 (0.1%)	714 (0.0%)
Kidney disease	CKD5 without dialysis or transplant	18,609 (1.4%)	105,376 (4.7%)	121,038 (7.4%)
	CKD5 with dialysis	238 (0.0%)	928 (0.0%)	812 (0.0%)
	CKD5 with transplant	225 (0.0%)	1,530 (0.1%)	1,132 (0.1%)

^†^Deprivation status: quintiles of UK IMD (2000).

^‡^Number of risk groups: count of QCovid risk groups.

^§^Number of previous tests: proxy for working in a high-risk occupation (e.g., healthcare worker).

BMI, body mass index; CKD, chronic kidney disease; COPD, chronic obstructive pulmonary disease; CVST, cerebral venous sinus thrombosis; IMD, index of multiple deprivation; SD, standard deviation; SLE, systemic lupus erythematosus; TIA, transient ischemic attack.

**Table 2:** Cohort summary statistics, Scotland.

Characteristic	Levels	Unvaccinated	One-dose ChAdOx1	One-dose BNT162b2
Total		1,837,555	1,743,343	829,038
Person years of follow-up		627,935	1,136,652	952,284
Incident CVST cases		9	34	13
Deaths		14,792 (0.80%)	12,485 (0.72%)	6,554 (0.79%)
Sex	Female	847,192 (46.1%)	900,591 (51.7%)	519,423 (62.7%)
	Male	990,363 (53.9%)	842,752 (48.3%)	309,615 (37.3%)
Age (years)	Mean (SD)	35.8 (13.6)	59 (15.3)	57.4 (16.6)
Age group (years)	18 to 64	1,756,236 (95.6%)	1,178,096 (67.6%)	462,906 (55.8%)
	65 to 79	47,113 (2.6%)	380,175 (21.8%)	329,739 (39.8%)
	80+	34,206 (1.9%)	185,072 (10.6%)	36,393 (4.4%)
Deprivation status^†^	1 (high)	393,735 (21.4%)	315,337 (18.1%)	152,365 (18.4%)
	2	363,509 (19.8%)	340,417 (19.5%)	164,078 (19.8%)
	3	345,606 (18.8%)	361,645 (20.7%)	166,194 (20.0%)
	4	345,959 (18.8%)	362,057 (20.8%)	176,278 (21.3%)
	5 (low)	364,644 (19.8%)	355,356 (20.4%)	165,303 (19.9%)
	Unknown	24,101 (1.3%)	8,531 (0.5%)	4,820 (0.6%)
Urban/rural index	1—large urban areas	779,728 (42.4%)	527,068 (30.2%)	246,393 (29.7%)
	2—other urban areas	592,533 (32.2%)	652,571 (37.4%)	334,968 (40.4%)
	3—accessible small towns	148,961 (8.1%)	178,437 (10.2%)	80,831 (9.7%)
	4—remote small towns	72,218 (3.9%)	98,686 (5.7%)	42,336 (5.1%)
	5—accessible rural	147,110 (8.0%)	179,672 (10.3%)	71,206 (8.6%)
	6—remote rural	72,905 (4.0%)	98,378 (5.6%)	48,484 (5.8%)
	Unknown	24,101 (1.3%)	8,531 (0.5%)	4,820 (0.6%)
Number of risk group^‡^	0	1,380,273 (75.1%)	812,869 (46.6%)	411,082 (49.6%)
	1	370,157 (20.1%)	519,923 (29.8%)	241,381 (29.1%)
	2	65,651 (3.6%)	238,303 (13.7%)	103,522 (12.5%)
	3	13,232 (0.7%)	100,913 (5.8%)	42,747 (5.2%)
	4	4,740 (0.3%)	43,232 (2.5%)	18,217 (2.2%)
	5+	3,502 (0.2%)	28,103 (1.6%)	12,089 (1.5%)
Number of previous tests^§^	0	1,521,965 (82.8%)	1,450,847 (83.2%)	599,480 (72.3%)
	1	237,779 (12.9%)	204,180 (11.7%)	114,003 (13.8%)
	2	46,071 (2.5%)	46,784 (2.7%)	33,299 (4.0%)
	3	11,350 (0.6%)	15,396 (0.9%)	14,173 (1.7%)
	4 to 9	11,842 (0.6%)	18,816 (1.1%)	27,847 (3.4%)
	10+	8,548 (0.5%)	7,320 (0.4%)	40,236 (4.9%)
Average household age	Mean (SD)	34.4 (13.8)	54.8 (17.8)	53.7 (18.5)
Number of people in household^¶^	1	545,823 (29.7%)	613,519 (35.2%)	259,370 (31.3%)
	2	404,714 (22.0%)	603,417 (34.6%)	298,158 (36.0%)
	3 to 5	784,498 (42.7%)	493,935 (28.3%)	236,711 (28.6%)
	6 to 10	82,513 (4.5%)	30,138 (1.7%)	15,493 (1.9%)
	11 to 30	8,569 (0.5%)	1,532 (0.1%)	6,669 (0.8%)
	31 to 100	6,255 (0.3%)	600 (0.0%)	10,967 (1.3%)
	101+	5,182 (0.3%)	202 (0.0%)	1,670 (0.2%)
BMI	Underweight	23,875 (1.3%)	17,726 (1.0%)	7,908 (1.0%)
	Normal weight	234,944 (12.8%)	214,888 (12.3%)	108,885 (13.1%)
	Overweight	1,422,006 (77.4%)	1,108,478 (63.6%)	519,249 (62.6%)
	Obese	156,730 (8.5%)	402,251 (23.1%)	192,996 (23.3%)
Smoking status	Ex-smoker	143,236 (7.8%)	297,503 (17.1%)	138,702 (16.7%)
	Nonsmoker	700,880 (38.1%)	669,699 (38.4%)	328,683 (39.6%)
	Smoker	299,528 (16.3%)	430,035 (24.7%)	192,753 (23.3%)
	Unknown	693,910 (37.8%)	346,106 (19.9%)	168,900 (20.4%)
Atrial fibrillation		6,353 (0.3%)	73,050 (4.2%)	29,466 (3.6%)
Asthma		224,636 (12.2%)	243,888 (14.0%)	109,010 (13.1%)
Blood cancer		1,772 (0.1%)	14,751 (0.8%)	5,541 (0.7%)
Heart failure		3,448 (0.2%)	33,261 (1.9%)	12,293 (1.5%)
Cerebral palsy		664 (0.0%)	4,334 (0.2%)	1,072 (0.1%)
Coronary heart disease		11,859 (0.6%)	133,205 (7.6%)	60,514 (7.3%)
Cirrhosis		3,483 (0.2%)	14,263 (0.8%)	6,204 (0.7%)
Congenital heart disease		3,586 (0.2%)	24,379 (1.4%)	10,277 (1.2%)
COPD		9,670 (0.5%)	90,514 (5.2%)	35,037 (4.2%)
Dementia		3,276 (0.2%)	17,779 (1.0%)	17,286 (2.1%)
Diabetes type 1		2,005 (0.1%)	14,889 (0.9%)	5,589 (0.7%)
Diabetes type 2		16,641 (0.9%)	167,766 (9.6%)	79,735 (9.6%)
Epilepsy		9,160 (0.5%)	43,185 (2.5%)	12,989 (1.6%)
Fracture		55,777 (3.0%)	97,098 (5.6%)	43,284 (5.2%)
Neurological disorder		1,433 (0.1%)	12,562 (0.7%)	4,561 (0.6%)
Parkinson disease		626 (0.0%)	6,061 (0.3%)	3,093 (0.4%)
Pulmonary hypertension		709 (0.0%)	6,523 (0.4%)	1,722 (0.2%)
Pulmonary rare		1,399 (0.1%)	16,060 (0.9%)	5,849 (0.7%)
Peripheral vascular disease		3,441 (0.2%)	28,820 (1.7%)	12,450 (1.5%)
Rheumatoid arthritis or SLE		3,414 (0.2%)	31,267 (1.8%)	12,947 (1.6%)
Respiratory cancer		1,218 (0.1%)	7,243 (0.4%)	2,552 (0.3%)
Severe mental illness		161,104 (8.8%)	263,637 (15.1%)	119,114 (14.4%)
Sickle cell disease		388 (0.0%)	1,905 (0.1%)	762 (0.1%)
Stroke/TIA		7,931 (0.4%)	80,030 (4.6%)	35,481 (4.3%)
Thrombosis or pulmonary embolus		8,086 (0.4%)	50,760 (2.9%)	18,625 (2.2%)
Care housing category	Care home	2,277 (0.1%)	2,806 (0.2%)	16,583 (2.0%)
	Homeless	2,271 (0.1%)	1,360 (0.1%)	342 (0.0%)
Learning disability or Down syndrome	Learning disability	24,695 (1.3%)	34,031 (2.0%)	9,551 (1.2%)
	Down syndrome	116 (0.0%)	1,193 (0.1%)	391 (0.0%)
Kidney disease	CKD5 without dialysis or transplant	7,558 (0.4%)	103,725 (5.9%)	41,568 (5.0%)
	CKD5 with dialysis	546 (0.0%)	4,335 (0.2%)	1,394 (0.2%)
	CKD5 with transplant	370 (0.0%)	3,107 (0.2%)	1,279 (0.2%)

^†^Deprivation status: quintiles of SIMD 2020.

^‡^Number of risk groups: count of QCovid risk groups.

^§^Number of previous tests: proxy for working in a high-risk occupation (e.g., healthcare worker).

^¶^Household information taken from September 2020.

BMI, body mass index; CKD, chronic kidney disease; COPD, chronic obstructive pulmonary disease; CVST, cerebral venous sinus thrombosis; SD, standard deviation; SIMD, Scottish Index of Multiple Deprivation; SLE, systemic lupus erythematosus; TIA, transient ischemic attack.

**Table 3:** Cohort summary statistics, Wales.

Characteristic	Levels	Unvaccinated	One-dose ChAdOx1	One-dose BNT162b2
Total		346,683	964,494	717,224
Person years of follow-up		205,886	541,057	402,723
Incident CVST cases		6	24	9
Deaths		7,741 (2.2%)	6,876 (0.7%)	1,388 (0.2%)
Sex	Male	197,387 (56.9%)	476,300 (49.4%)	329,736 (46.0%)
	Female	149,296 (43.1%)	488,194 (50.6%)	387,488 (54.0%)
Age (years)	Mean (SD)	36.27 (17.10)	58.49 (15.94)	44.82 (19.60)
Age group (years)	18 to 64	274,386 (91.3%)	636,502 (66.0%)	538,559 (75.9%)
	65 to 79	17,389 (5.8%)	209,653 (21.7%)	158,915 (22.4%)
	80+	8,739 (2.9%)	117,968 (12.2%)	12,278 (1.7%)
Deprivation status^†^	1 (most deprived)	94,765 (27.3%)	183,424 (19.0%)	143,271 (20.0%)
	2	74,747 (21.6%)	195,260 (20.2%)	146,938 (20.5%)
	3	69,401 (20.0%)	190,358 (19.7%)	131,847 (18.4%)
	4	53,523 (15.4%)	191,504 (19.9%)	131,130 (18.3%)
	5 (least deprived)	54,247 (15.6%)	203,948 (21.1%)	164,038 (22.9%)
Number of risk groups^‡^	1	129,012 (37.2%)	181,916 (18.9%)	211,942 (29.6%)
	2	122,262 (35.3%)	305,529 (31.7%)	256,138 (35.7%)
	3	60,691 (17.5%)	239,308 (24.8%)	150,040 (20.9%)
	4	22,745 (6.6%)	129,906 (13.5%)	61,613 (8.6%)
	5+	11,973 (3.5%)	107,835 (11.2%)	37,491 (5.2%)
Number of previous tests^§^	0	289,816 (83.6%)	787,529 (81.7%)	525,062 (73.2%)
	1	39,404 (11.4%)	122,007 (12.6%)	123,853 (17.3%)
	2	9,074 (2.6%)	28,177 (2.9%)	32,648 (4.6%)
	3	2,676 (0.8%)	9,090 (0.9%)	9,544 (1.3%)
	4 to 9	3,371 (1.0%)	11,425 (1.2%)	10,037 (1.4%)
Average household age	Mean (SD)	36.12 (15.09)	52.28 (18.83)	42.83 (18.64)
Number of people in household	1	35,087 (10.1%)	164,573 (17.1%)	77,093 (10.8%)
	2	59,280 (17.1%)	322,717 (33.5%)	190,182 (26.5%)
	3 to 5	191,646 (55.4%)	413,605 (42.9%)	389,868 (54.4%)
	6 to 10	52,028 (15.0%)	47,698 (4.9%)	53,992 (7.5%)
	11 to 30	5,817 (1.7%)	10,467 (1.1%)	4,431 (0.6%)
	31 to 100	1,164 (0.3%)	4,356 (0.5%)	882 (0.1%)
	101+	830 (0.2%)	183 (0.0%)	408 (0.1%)
BMI	Normal weight	137,044 (39.8%)	238,075 (24.7%)	214,268 (29.9%)
	Obese	75,615 (21.9%)	374,711 (38.9%)	237,832 (33.2%)
	Overweight	103,442 (30.0%)	325,633 (33.8%)	237,648 (33.2%)
	Underweight	28,634 (8.3%)	25,261 (2.6%)	26,418 (3.7%)
Smoking status	Ex-smoker	37,314 (10.8%)	231,039 (24.0%)	127,107 (17.7%)
	Nonsmoker	140,892 (40.6%)	512,280 (53.1%)	402,210 (56.1%)
	Smoker	98,437 (28.4%)	201,499 (20.9%)	136,068 (19.0%)
	Unknown	70,040 (20.2%)	19,676 (2.0%)	51,839 (7.2%)
Atrial fibrillation		3,005 (0.9%)	43,976 (4.6%)	15,274 (2.1%)
Asthma		45,591 (13.2%)	147,572 (15.3%)	110,270 (15.4%)
Blood cancer		699 (0.2%)	7,116 (0.7%)	2,886 (0.4%)
Heart failure		1,816 (0.5%)	22,248 (2.3%)	6,879 (1.0%)
Cirrhosis		586 (0.2%)	4,903 (0.5%)	1,889 (0.3%)
Congestive heart disease		4,482 (1.3%)	61,338 (6.4%)	22,283 (3.1%)
COPD		3,794 (1.1%)	44,984 (4.7%)	16,158 (2.3%)
Dementia		1,438 (0.4%)	13,155 (1.4%)	2,540 (0.4%)
Diabetes type I		714 (0.2%)	6,034 (0.6%)	1,628 (0.2%)
Diabetes type II		8,081 (2.3%)	108,610 (11.3%)	36,764 (5.1%)
Epilepsy		3,295 (1.0%)	18,776 (1.9%)	5,136 (0.7%)
Fracture		11,205 (3.2%)	40,808 (4.2%)	26,066 (3.6%)
Neurological disorder		439 (0.1%)	4,260 (0.4%)	1,154 (0.2%)
Parkinson disease		313 (0.1%)	3,845 (0.4%)	1,312 (0.2%)
Pulmonary hypertension		254 (0.1%)	2,550 (0.3%)	904 (0.1%)
Pulmonary rare		528 (0.2%)	6,287 (0.7%)	2,755 (0.4%)
Peripheral vascular disease		1,107 (0.3%)	12,368 (1.3%)	4,547 (0.6%)
Rheumatoid arthritis		1,376 (0.4%)	14,754 (1.5%)	6,454 (0.9%)
Respiratory cancer		495 (0.1%)	3,620 (0.4%)	1,621 (0.2%)
Severe mental illness		42,890 (12.4%)	153,083 (15.9%)	87,343 (12.2%)
Stroke		3,175 (0.9%)	39,032 (4.0%)	12,706 (1.8%)
Thrombosis or pulmonary embolus		3,468 (1.0%)	30,726 (3.2%)	10,296 (1.4%)
Care housing category	Care home	1,150 (0.3%)	7,729 (0.8%)	1,375 (0.2%)
	Homeless	2,308 (0.7%)	2,431 (0.3%)	1,230 (0.2%)
Learning disability or Down syndrome	Learning disability	7,232 (2.1%)	16,250 (1.7%)	9,397 (1.3%)
	Down syndrome	25 (0.0%)	195 (0.0%)	74 (0.0%)
Kidney disease	CKD5 without dialysis or transplant	277 (0.1%)	2,711 (0.3%)	802 (0.1%)
	CKD5 with transplant	69 (0.0%)	921 (0.1%)	364 (0.1%)
	CKD5 with dialysis	47 (0.0%)	304 (0.0%)	61 (0.0%)

^†^Deprivation status: WIMD 2020.

^‡^Number of risk groups: Individual QCovid.

^§^Number of previous tests: Proxy for working in a high-risk occupation (e.g., healthcare worker).

BMI, body mass index; CKD, chronic kidney disease; COPD, chronic obstructive pulmonary disease; CVST, cerebral venous sinus thrombosis; SD, standard deviation; SLE, systemic lupus erythematosus; TIA, transient ischemic attack; WIMD, Welsh index of multiple deprivation.

**Table 4:** Number of events and IRRs for CVST following first dose vaccination with ChAdOx1 and BNT162b2.

Time period	Number of events	IRR (95% CI)
ChAdOx1		
Reference	45	1
Prerisk	9	1.29 (0.63 to 2.63)
Risk	27	1.93 (1.20 to 3.11)
BNT162b2		
Reference	29	1
Prerisk	<5	0.89 (0.31 to 2.52)
Risk	7	0.78 (0.34 to 1.77)

Event counts of <5 have been suppressed in accordance with disclosure control principles implemented by the data controllers. With the day of vaccination as day 0, the reference period was day −104 to day −14. The prerisk period was day −14 to day 0. The risk period was day 0 to day 28.

CI, confidence interval; CVST, cerebral venous sinus thrombosis; IRR, incidence rate ratio.

We sought to carry out a post hoc sensitivity analysis focusing on CVST events identified in secondary care records only; however, there were too few events to permit useful estimation of IRRs. We carried out a post hoc sensitivity analysis excluding anyone who died within 90 days of their CVST event. The only estimate that changed from our main analysis was that for the risk period following vaccination with ChAdOx1, the IRR was 1.79 (95% CI 1.10 to 2.91) (S6 File).

Within each nation, we plotted histograms of event counts in the observation period in order to explore the suitability of the prerisk period to account for event-dependent exposure to vaccination. There appeared to be a reduction in events in the 2-week prerisk period, but no obvious reduction outside of that time period. We are unable to share these histograms due to the statistical disclosure principles enacted by the TREs that hosted this analysis.

## Discussion

Our pooled SCCS analysis of national datasets from England, Scotland, and Wales found an elevated risk of CVST in the 4-week period following vaccination with ChAdOx1. We did not find an association between BNT162b2 and CVST.

There have hitherto been few population-based studies estimating the association between COVID-19 vaccines and CVST. Such analyses are made challenging by the extreme rarity of CVST. Our study corroborates previous observed–expected analyses from the EMA [7], the Mayo Clinic Health System [8], a population-based study in Denmark and Norway [9], and a SCCS using the QResearch database in England [10]. Our results are broadly consistent with the latter study [10], as would be expected due to the similar study design and population. Our estimated RRs are, however, notably smaller than risk ratios reported in [7–9]. This is likely due to the fact that we conducted a SCCS that implicitly controls for variables that are constant over the observation period, as opposed to an observed–expected analysis.

A limitation in the SCCS analysis is the assumption that occurrence of an event does not affect subsequent exposure [25]. CVST is a serious adverse event that can be life threatening. Therefore, the assumption of event-independent exposure may not have been satisfied. This could have caused a selection effect where individuals who were more likely to have a CVST event were less likely to be vaccinated and thus less likely to be included in our analysis. A large multinational study of prognosis for CVST found a death rate of 4.3% and a death or dependency rate of 18.9% at hospital discharge, where dependency was defined as a score of >2 on the modified Rankin scale [26]. On the other hand, among the JCVI priority groups for vaccination are “clinically extremely vulnerable individuals” (group 4), and “all individuals aged 16 to 64 years with underlying health conditions which put them at higher risk of serious disease and mortality” (group 6) (S2 File). It is possible that the vaccination programme created a selection effect for vaccination of people more prone to CVST events.

In order to explore the validity of the assumption of event-independent exposure, we carried out a sensitivity analysis excluding those who died within 90 days of their event. This had a small effect on the IRR for the risk period following vaccination with ChAdOx1, with all other estimates unchanged. We also included a prerisk period in the SCCS study design to account for the possibility that occurrence of a CVST event affected subsequent vaccination and plotted histograms of event counts over time in order to assess its suitability. Our statistical analysis plan included a case–control analysis, which may have been useful to further explore this assumption. However, this would have required sharing more detailed data between TREs, which was not possible under the permissions in place.

We estimated IRRs as opposed to RRs. Estimating RRs with pooled data across the 3 nations could be achieved if we were able to share individual-level data. However, this was not permitted under the statistical disclosure rules implemented by the data controllers. We do not believe that the estimates for RRs and IRRs would be significantly different because of the rarity of CVST events.

We plan to extend our analysis to mRNA-1273 and to second and booster dose vaccinations. To date, there have been very few studies that have estimated the association between COVID-19 vaccine and CVST events using large-scale nationally representative datasets [7–9]. Although we had access to a large, combined cohort, there were still relatively few events. Further evidence corroborating our results is required.

In conclusion, we found an increased risk of CVST following first dose vaccination with ChAdOx1. We did not find an increased risk following first dose vaccination with BNT162b2. This evidence may be useful in risk–benefit evaluations for vaccine-related policies and in providing quantification of risks associated with vaccination to the general public.

## Supporting information

S1 File STROBE and RECORD checklists. RECORD, REporting of studies Conducted using Observational Routinely-collected Data; STROBE, STrengthening the Reporting of OBservational studies in Epidemiology.
(DOCX)

S2 File Vaccine priority groups. Context of vaccine roll-out in the UK: JCVI COVID-19 vaccination priority group list. COVID-19, Coronavirus Disease 2019; JCVI, Joint Committee on Vaccination and Immunisation.
(DOCX)

S3 File Statistical analysis plan. (DOCX)

S4 File Code lists. Read Codes and SNOMED CT codes for CVST. CVST, cerebral venous sinus thrombosis.
(DOCX)

S5 File Data pooling procedure. (DOCX)

S6 File Sensitivity analysis. (DOCX)

## Abbreviations

CHI: Community Health Index
CI: confidence interval
COVID-19: Coronavirus Disease 2019
CVST: cerebral venous sinus thrombosis
EAVE: Early Assessment of Vaccine and antiviral Effectiveness
EMA: European Medicines Agency
ICD-10: International Classification of Diseases-10th Revision
IGRP: Information Governance Review Panel
IRR: incidence rate ratio
JCVI: Joint Committee on Vaccination and Immunisation
RECORD: REporting of studies Conducted using Observational Routinely-collected Data
RR: rate ratio
SCCS: self-controlled case series
TRE: trusted research environment
