# Supplementary material for: First dose ChAdOx1 and BNT162b2 COVID-19 vaccinations and cerebral venous sinus thrombosis: A pooled self-controlled case series study of 11.6 million individuals in England, Scotland, and Wales
Source: PLoS Med. 2022 Feb 22;19(2):e1003927. doi: 10.1371/journal.pmed.1003927 (PMC8863261; doi:10.1371/journal.pmed.1003927)
Supplement: S1 File — RECORD, REporting of studies Conducted using Observational Routinely-collected Data; STROBE, STrengthening the Reporting of OBservational studies in Epidemiology. (DOCX) [file pmed.1003927.s001.docx]

**S1 Checklist. STROBE and RECORD checklists**

|  | **Item No.** | **STROBE items** | **Location in manuscript where items are reported** | **RECORD items** | **Location in manuscript where items are reported** |
| --- | --- | --- | --- | --- | --- |
| **Title and abstract** | | | | | |
|  | 1 | (a) Indicate the study’s design with a commonly used term in the title or the abstract (b) Provide in the abstract an informative and balanced summary of what was done and what was found | a) Title page, ‘First dose ChAdOx1 and BNT162b2 COVID-19 vaccinations and cerebral venous sinus thrombosis: pooled self-controlled case series of UK datasets’  b) Introduction, ‘We carried out a self-controlled case series (SCCS) analysis of incident CVST events following first dose vaccination with ChAdOx1 and BNT162b2...’ | RECORD 1.1: The type of data used should be specified in the title or abstract. When possible, the name of the databases used should be included.  RECORD 1.2: If applicable, the geographic region and timeframe within which the study took place should be reported in the title or abstract.  RECORD 1.3: If linkage between databases was conducted for the study, this should be clearly stated in the title or abstract. | 1.1, 1.2) Title page, ‘First dose ChAdOx1 and BNT162b2 COVID-19 vaccinations and cerebral venous sinus thrombosis: pooled self-controlled case series of UK datasets’  1.3) Abstract, ‘ Counts of CVST cases were collated from datasets consisting of linked primary care, secondary care, mortality and virological testing data in each of England, Scotland and Wales, with a combined cohort of ~12.20 million people.’ |
| **Introduction** | | | | | |
| Background rationale | 2 | Explain the scientific background and rationale for the investigation being reported |  |  | Introduction, ‘There have been concerns over possible associations between some COVID-19 vaccines and haematological and vascular adverse events, including, in particular, cerebral venous sinus thrombosis (CVST) following ChAdOx1 nCoV-19...’ |
| Objectives | 3 | State specific objectives, including any prespecified hypotheses |  |  | Introduction, ‘The aim of this study was to investigate possible associations between COVID-19 vaccines and CVST.’ |
| **Methods** | | | | | |
| Study Design | 4 | Present key elements of study design early in the paper |  |  | Study design and population |
| Setting | 5 | Describe the setting, locations, and relevant dates, including periods of recruitment, exposure, follow-up, and data collection |  |  | Study design and population, ‘The datasets consisted of linked primary care, secondary care, mortality and virological testing data stored in secure trusted research environments (TREs) in each of England, Scotland and Wales (Fig 1)….’ |
| Participants | 6 | *(a) Cohort study* - Give the eligibility criteria, and the sources and methods of selection of participants. Describe methods of follow-up  *Case-control study* - Give the eligibility criteria, and the sources and methods of case ascertainment and control selection. Give the rationale for the choice of cases and controls  *Cross-sectional study* - Give the eligibility criteria, and the sources and methods of selection of participants  *(b) Cohort study* - For matched studies, give matching criteria and number of exposed and unexposed  *Case-control study* - For matched studies, give matching criteria and the number of controls per case | Study design and population, ‘Anyone under the age of 16 at the date of event was excluded.’ | RECORD 6.1: The methods of study population selection (such as codes or algorithms used to identify subjects) should be listed in detail. If this is not possible, an explanation should be provided.  RECORD 6.2: Any validation studies of the codes or algorithms used to select the population should be referenced. If validation was conducted for this study and not published elsewhere, detailed methods and results should be provided.  RECORD 6.3: If the study involved linkage of databases, consider use of a flow diagram or other graphical display to demonstrate the data linkage process, including the number of individuals with linked data at each stage. | 6.1) Study design and population, ‘Anyone under the age of 16 at the date of event was excluded.’  6.2) NA  6.3) Fig 1 |
| Variables | 7 | Clearly define all outcomes, exposures, predictors, potential confounders, and effect modifiers. Give diagnostic criteria, if applicable. | Exposure,  Outcome | RECORD 7.1: A complete list of codes and algorithms used to classify exposures, outcomes, confounders, and effect modifiers should be provided. If these cannot be reported, an explanation should be provided. | S2 and S3 Table |
| Data sources/ measurement | 8 | For each variable of interest, give sources of data and details of methods of assessment (measurement).  Describe comparability of assessment methods if there is more than one group |  |  | Fig 1  Outcome, ‘The outcome of interest were incident cases of CVST in the observation period. SNOMED codes were used to identify CVST events recorded in primary care electronic health records in England S2 Table and Read codes (Version 2) were used in Scotland and Wales S3 Table.’ |
| Bias | 9 | Describe any efforts to address potential sources of bias |  |  | Disucssion, ‘A limitation in the SCCS analysis is the assumption that occurrence of an event does not affect subsequent exposure...‘ |
| Study size | 10 | Explain how the study size was arrived at |  |  | NA |
| Quantitative variables | 11 | Explain how quantitative variables were handled in the analyses. If applicable, describe which groupings were chosen, and why |  |  | Statistical Analysis, ‘Aggregate level data with counts of incident cases in the reference, pre-risk and risk periods stratified by vaccine type received were collated in each nation...’ |
| Statistical methods | 12 | (a) Describe all statistical methods, including those used to control for confounding  (b) Describe any methods used to examine subgroups and interactions  (c) Explain how missing data were addressed  (d) *Cohort study* - If applicable, explain how loss to follow-up was addressed  *Case-control study* - If applicable, explain how matching of cases and controls was addressed  *Cross-sectional study* - If applicable, describe analytical methods taking account of sampling strategy  (e) Describe any sensitivity analyses |  |  | Statistical analysis |
| Data access and cleaning methods |  | .. | NA | RECORD 12.1: Authors should describe the extent to which the investigators had access to the database population used to create the study population.  RECORD 12.2: Authors should provide information on the data cleaning methods used in the study. | Statistical Analysis, ‘Analysts in each country had full access to that country’s data.’ |
| Linkage |  | .. |  | RECORD 12.3: State whether the study included person-level, institutional-level, or other data linkage across two or more databases. The methods of linkage and methods of linkage quality evaluation should be provided. | Study design and population, ‘These data were deterministically linked using unique patient identifiers – NHS number in England, and Community Health Index (CHI) number in Scotland...’ |
| **Results** | | | | | |
| Participants | 13 | (a) Report the numbers of individuals at each stage of the study (*e.g.*, numbers potentially eligible, examined for eligibility, confirmed eligible, included in the study, completing follow-up, and analysed)  (b) Give reasons for non-participation at each stage.  (c) Consider use of a flow diagram | pg8, Table 1 | RECORD 13.1: Describe in detail the selection of the persons included in the study (*i.e.,* study population selection) including filtering based on data quality, data availability and linkage. The selection of included persons can be described in the text and/or by means of the study flow diagram. | Introduction, ‘In our previous analysis exploring COVID-19 vaccine associations with thrombocytopenic, thromboembolic and hemorrhagic events using Scottish national data, there were insufficient CVST events to undertake a statistical analysis [19]. We were not able to reliably estimate the association between COVID-19 vaccines and CVST with any individual country-specific dataset due to the low number of events. In order to address this, we pooled incident CVST cases from each of the datasets and carried out a SCCS analysis to estimate incident rate ratios (IRRs) for CVST in those who had received the first dose of ChAdOx1 or BNT162b2 vaccines.’ |
| Descriptive data | 14 | (a) Give characteristics of study participants (*e.g.*, demographic, clinical, social) and information on exposures and potential confounders  (b) Indicate the number of participants with missing data for each variable of interest  (c) *Cohort study* - summarise follow-up time (*e.g.*, average and total amount) |  |  | Table 1 |
| Outcome data | 15 | *Cohort study* - Report numbers of outcome events or summary measures over time  *Case-control study* - Report numbers in each exposure category, or summary measures of exposure  *Cross-sectional study* - Report numbers of outcome events or summary measures | 16 |  | Table 2 |
| Main results | 16 | (a) Give unadjusted estimates and, if applicable, confounder-adjusted estimates and their precision (e.g., 95% confidence interval). Make clear which confounders were adjusted for and why they were included  (b) Report category boundaries when continuous variables were categorized  (c) If relevant, consider translating estimates of relative risk into absolute risk for a meaningful time period |  |  | Table 2 |
| Other analyses | 17 | Report other analyses done—e.g., analyses of subgroups and interactions, and sensitivity analyses |  |  | NA |
| **Discussion** | | | | | |
| Key results | 18 | Summarise key results with reference to study objectives |  |  | Discussion, ‘Our novel pooled SCCS analysis of national datasets from England, Scotland and Wales found an elevated risk of CVST in the four-week period following vaccination with ChAdOx1. We did not find an association between BNT162b2 and CVST.’ |
| Limitations | 19 | Discuss limitations of the study, taking into account sources of potential bias or imprecision. Discuss both direction and magnitude of any potential bias |  | RECORD 19.1: Discuss the implications of using data that were not created or collected to answer the specific research question(s). Include discussion of misclassification bias, unmeasured confounding, missing data, and changing eligibility over time, as they pertain to the study being reported. | Discussion, ‘A limitation in the SCCS analysis is the assumption that occurrence of an event does not affect subsequent exposure...’ |
| Interpretation | 20 | Give a cautious overall interpretation of results considering objectives, limitations, multiplicity of analyses, results from similar studies, and other relevant evidence |  |  | Discussion, ‘In conclusion, we found an increased risk of CVST following first-dose vaccination with ChAdOx1. We did not find an increased risk following first dose vaccination with BNT162b2.’ |
| Generalisability | 21 | Discuss the generalisability (external validity) of the study results |  |  | Discussion, ‘Although we had access to a large combined cohort, there were still relatively few events. Further evidence corroborating our results is required.’ |
| **Other Information** | | | | | |
| Funding | 22 | Give the source of funding and the role of the funders for the present study and, if applicable, for the original study on which the present article is based |  |  | Financial disclosure section of submission form |
| Accessibility of protocol, raw data, and programming code |  | .. |  | RECORD 22.1: Authors should provide information on how to access any supplemental information such as the study protocol, raw data, or programming code. | Data availability statement |
