# Supplementary material for: First dose ChAdOx1 and BNT162b2 COVID-19 vaccinations and cerebral venous sinus thrombosis: A pooled self-controlled case series study of 11.6 million individuals in England, Scotland, and Wales
Source: PLoS Med. 2022 Feb 22;19(2):e1003927. doi: 10.1371/journal.pmed.1003927 (PMC8863261; doi:10.1371/journal.pmed.1003927)
Supplement: S2 File — Context of vaccine roll-out in the UK: JCVI COVID-19 vaccination priority group list. COVID-19, Coronavirus Disease 2019; JCVI, Joint Committee on Vaccination and Immunisation. (DOCX) [file pmed.1003927.s002.docx]

**S2 Vaccine priority groups. Context of vaccine roll-out in UK: Joint Committee on Vaccination and Immunisation (JCVI) COVID-19 vaccination priority group list.**

| **Order of priority** | **Priority Group** |
| --- | --- |
| **Phase 1** |  |
| 1 | Residents in a care home for older adults and their carers |
| 2 | All those 80 years of age and over and frontline health and social care workers |
| 3 | All those 75 years of age and over |
| 4 | All those 70 years of age and over and clinically extremely vulnerable individuals |
| 5 | All those 65 years of age and over |
| 6 | All individuals aged 16 years to 64 years with underlying health conditions which put them at higher risk of serious disease and mortality |
| 7 | All those 60 years of age and over |
| 8 | All those 55 years of age and over |
| 9 | All those 50 years of age and over |
| Phase 2 |  |
| 1 | All those aged 40-49 years |
| 2 | All those aged 30-39 years |
| 3 | All those aged 18-29 years |
| Note: The vaccine roll-out strategy has been determined by an independent UK-wide body, namely the Joint Commission on Vaccinations and Immunisation (JCVI),^1^ which has prioritised vaccinations to adults on the basis of assessing the risk of serious COVID-19 outcomes, in particular hospitalisations and deaths.^1^ The offer of vaccination during phase 2 is age-based starting with the oldest adjust first and proceeding in the order as listed.^2^  Because of the different storage requirements for the two vaccines, GPs have administered the ChAdOx1 (Oxford-AstraZeneca) vaccine and vaccine centres have mainly administered the BNT162b2 (Pfizer-BioNTech) vaccine. Guided by JCVI priorities, GPs began by focusing their efforts on: a) the mobile elderly who they vaccinated in their general practice surgeries; and b) care home residents affiliated with general practices. Vaccination centres began with focusing on health and social care providers before extending to other JCVI priority groups.  1. Joint Committee on Vaccination and Immunisation. Priority groups for coronavirus (COVID-19) vaccination: advice from the JCVI, 30 December 2020. Available from: <https://www.gov.uk/government/publications/priority-groups-for-coronavirus-covid-19-vaccination-advice-from-the-jcvi-30-december-2020>  2. Joint Committee on Vaccination and Immunisation. JCVI interim statement on phase 2 of the COVID-19 vaccination programme. Available from: <https://www.gov.uk/government/publications/priority-groups-for-phase-2-of-the-coronavirus-covid-19-vaccination-programme-advice-from-the-jcvi/jcvi-interim-statement-on-phase-2-of-the-covid-19-vaccination-programme> | |
