# Supplementary material for: First dose ChAdOx1 and BNT162b2 COVID-19 vaccinations and cerebral venous sinus thrombosis: A pooled self-controlled case series study of 11.6 million individuals in England, Scotland, and Wales
Source: PLoS Med. 2022 Feb 22;19(2):e1003927. doi: 10.1371/journal.pmed.1003927 (PMC8863261; doi:10.1371/journal.pmed.1003927)

**S3 Statistical analysis plan**

Analysis Plan for Programmatic evaluation of COVID-19 vaccine safety using linked UK national data

| **Full Project Title** | DaCVaP |
| --- | --- |
| **Version Number** | V1 |
| **Previous Versions** | N/A |
| **Effective Date** | 26 April 2021 |
| **Analyst(s)** | *Chris Robertson (CR), Eleftheria Vasileiou (EV) |
| **Co-authors** |  |
| **Target journal** |  |

*Corresponding author

| **Version History** | | | |
| --- | --- | --- | --- |
| **Version** | **Date** | **Author** | **Notes** |
| V1 | 26.04.2021 | CR, EV | First version sent to team |
|  |  |  |  |
|  |  |  |  |

Contents

[1 Introduction 3](#_gjdgxs)

[2 Aims and objectives 3](#_30j0zll)

[2.1 Aims 3](#_1fob9te)

[2.2 Objectives 3](#_3znysh7)

[3 Study Design 3](#_2et92p0)

[3.1 Study design 3](#_tyjcwt)

[3.2 Setting 3](#_3dy6vkm)

[3.3 Population 3](#_1t3h5sf)

[3.4 Data sources 3](#_4d34og8)

[3.5 Inclusion/exclusion criteria 4](#_2s8eyo1)

[3.6 Sample size calculation 4](#_17dp8vu)

[4 Data and data validation 4](#_3rdcrjn)

[4.1 Data variables available 4](#_26in1rg)

[4.2 Constructed variables 6](#_lnxbz9)

[4.3 Consistency and error checking 6](#_35nkun2)

[5 Statistical analyses 6](#_1ksv4uv)

[5.1 Objective a. Investigate the safety of Pfizer-BioNTech, Oxford-AstraZeneca and Moderna vaccines against mild-to-moderate and severe adverse events following immunisation, respectively. 6](#_44sinio)

[5.1.1 Exposures of interest 6](#_2jxsxqh)

[5.1.2 Outcomes of interest 6](#_z337ya)

[5.1.3 Potential confounders 6](#_3j2qqm3)

[5.1.4 Potential effect modifiers 6](#_1y810tw)

[5.1.5 Analytical techniques 7](#_4i7ojhp)

[5.1.6 Sub-group analysis 7](#_2xcytpi)

[5.1.7 Corrections for multiple testing 7](#_1ci93xb)

[5.1.8 Sensitivity analysis 7](#_3whwml4)

[5.1.9 Other analysis 7](#_2bn6wsx)

[5.2 Missing data 7](#_qsh70q)

[5.3 Statistical software 8](#_3as4poj)

[6 Reporting results 9](#_1pxezwc)

[6.1 Reporting guidelines and conventions 9](#_49x2ik5)

[6.2 Dissemination 9](#_2p2csry)

[7 References 10](#_147n2zr)

[8 Appendix 10](#_3o7alnk)

# Introduction

The aim of this analysis is to investigate the safety of COVID-19 vaccines that are currently licensed in the UK (Pfizer-BioNTech, Oxford-AstraZeneca, Moderna) using data from 4 centres in the UK – academic groups linked to public health agencies in England, Northern Ireland, Scotland and Wales. Within each centre a data platform containing pseudonymised, linked data from GP records, vaccination records, secondary care, and virological testing will be used to analyse the data using a common analysis plan. These results will then be combined in a meta-analysis. All data and analyses will be hosted in secure research environments in each UK nation.

# Aims and objectives

## Aims

To study safety of Pfizer-BioNTech, Oxford-AstraZeneca and Moderna COVID-19 vaccines across the UK.

## Objectives

We seek to:

1. Investigate the safety of Pfizer-BioNTech, Oxford-AstraZeneca and Moderna vaccines (both first and second doses) against mild-to-moderate and severe adverse events following vaccination in each UK nation, and combine the results in a meta-analysis.

# Study Design

## Study design

Our primary analysis will use self-controlled case series for mild-to-moderate adverse events, and nested case-control studies for severe adverse events or where exposure is potentially event-dependent.

## Setting

UK

## Population

Individuals registered in GP practices across the UK.

## Data sources

- Primary care data: Routinely collected records from general practices (GP) containing information on demographics, clinical history, vaccination status and vaccination side-effects.
- Vaccination data: For England, GPs and the National Immunisation Management Service (NIMS). For Northern Ireland, GPs and Health and Social Care (HSC) Trusts. For Scotland, GPs and Turas Vaccination Management Tool (TVMT). For Wales, COVID Vaccination Data (CVVD) from the Welsh Immunisation System (WIS).
- Secondary care data: For England, hospital data are held in two forms: Hospital Episode Statistics (HES), which is the long-term validated record; and Secondary Uses Services (SUS) which is an extract of contemporary operational data that becomes HES after validation. For Northern Ireland, hospital admissions via the Admissions and discharge dataset. For Scotland, hospital admissions through the Scottish Morbidity Record (SMR) 01 and Rapid Preliminary Inpatient Data (RAPID). For Wales, inpatient hospital admissions through the Patient Episode Database for Wales (PEDW).
- Mortality data: For England, deaths from NHS Digital and Office for National Statistics (ONS). For Northern Ireland, deaths via National Health Applications and Infrastructure Services (NHAIS). For Scotland, deaths from the National Records of Scotland (NRS) database. For Wales, deaths data will derive from the National Population Spine (Welsh Demographic Service – WDSD), ONS death data (ADDE and ADDD) and a national NHS master patient index record (Consolidated Death Data Source – CDDS) - all which have been cleaned into a consolidated mortality data source C19_COHORT20_MORTALITY.
- Laboratory data: For England, testing data will derive from NHS Digital. For Northern Ireland, the Pillar 1 dataset from Laboratory Information System (LIS) and Pillar 2 dataset from NHS Digital. For Scotland, RT-PCR SARS-CoV-2 test data, available through the Electronic Communication of Surveillance in Scotland (ECOSS) database. For Wales, data is available for Pillar 1 and 2 from all NHS and private laboratories within the PATD data source.

## Inclusion/exclusion criteria

Inclusion criteria:

All participants eligible to receive a vaccination.

Exclusion criteria:

Age 17 or less at the start date of the study cohort.

## Sample size calculation

The number of COVID-10 vaccines doses required to detect a relative risk of 5.0 is at least 10,000 doses for a relatively common adverse outcome (e.g., myocardial infraction with a background incidence rate of 1400 per 100,000 person years in men older than 85 years old) and more than a million doses are needed to detect a relative risk of 1.5 for a rare adverse outcome (e.g., myocardial infraction with a background incidence rate of 28 per 100,000 person years among those 18-34 years).[1, 2]

# Data and data validation

## Data variables available

Table 1 lists the groupings of variables available for this study by data source. Exposure data are described in the Vaccinations category. Outcome data are described in the primary care, secondary care and mortality data categories. The rest of the categories contain data on potential confounding variables and effect modifiers.

**Table 1: Data items and data sources**

| **Data item** | **England** | **Northern Ireland** | **Scotland** | **Wales** |
| --- | --- | --- | --- | --- |
| Exposures | | | | |
| Pfizer-BioNTech vaccine | GP, NIMS | VMS | GP, TVMT/PHS, SIRS | CVVD |
| Oxford-AstraZeneca vaccine | GP, NIMS | VMS | GP, TVMT/PHS, SIRS | CVVD |
| Moderna vaccine | GP, NIMS | VMS | GP, TVMT/PHS, SIRS | CVVD |
| Laboratory confirmed SARS-CoV-2 infection | GP, Pillar 1&2  SGSS, PHE | Pillar 1 and Pillar 2 | ECOSS | PATD (Pillar 1, 2, & 3 data from all NHS and private labs), CVLF testing and results data |
| Outcomes | | | | |
| COVID-19 related GP consultation | GP | NA | GP | WLGP |
| COVID-19 related emergency department consultation | GP, ECDS | Symphony, NIRAES | SMR01 | EDDD & EDDS |
| COVID-19 related hospital admission | GP, SUS | Admissions and discharge dataset | SMR01 | PEDW |
| COVID-19 related ICU admission | GP, CHESS | Admissions and discharge dataset | SICSAG | CDDS, ICCD & ICNC |
| COVID-19 related death | GP, ONS, SSRS | NHAIS | NRS | ADDE & ADDE (ONS mortality), CDDS & WDSD |
| Secondary SARS-CoV-2 infection due to household transmission | RCGP RSC household key | Pillar 1 and Pillar 2 dataset | ECOSS | PATD (Pillar 1, 2, & 3 data from all NHS and private labs), CVLF testing and results data |
| Maternity outcomes | GP, MSDS | NIMATS | COPS study | ADBE (ONS births), MIDS & NCCH |
| Patient characteristics & confounders | | | | |
| Age | GP | NHAIS | GP | C19_COHORT20 |
| Sex | GP | NHAIS | GP | C19_COHORT20 |
| Socio-economic status | Post code to IMD | NHAIS | GP | C19_COHORT20 |
| Ethnicity | GP, SUS | VMS | Census 2011 | National ethnicity spine (made up of 20 EHR data sources and the ONS Census 2011) |
| Underlying medical condition | GP | EPD | GP | GP, WLGP, PEDW, CVSP |
| Type of settlement (urban/rural) | GP | NHAIS | GP | C19_COHORT20 |
| Type of settlement (e.g., private home, care home or social housing) | GP | NHAIS | GP | C19_COHORT20, CARE |
| Smoking status | GP | NA | GP | WLGP |
| Body Mass Index (BMI) | GP | NA | GP | WLGP |
| Prescribed medications | GP | EPD | GP, PIS, HEPMA | WLGP, WDDS |
| Other non-COVID-19 vaccines (e.g., influenza, pneumococcal) | GP | VMS | GP | WLGP, NCCH |
| Occupation (e.g., healthcare workers, frontline workers, essential workers) | GP where recorded | Pillar 1 and 2 | To be confirmed | HWRA, SWAC |
| History of healthcare utilisation (e.g., GP consultations, hospital admissions) | GP, SUS | Admissions and discharge dataset | GP, SMR01 | PEDW, WLGP |
| Annual District Birth Extract (ONS Births) (ADBE); Annual District Death Extract (ONS Deaths) (ADDE); COVID Vaccine Data (CVVD); COVID-19 Hospitalisation in England Surveillance System (CHESS); COVID-19 in Pregnancy in Scotland (COPS); COVID-19 Second Generation Surveillance System (SGSS); COVID-19 Shielded People list (CVSP); Critical Care DataSet (CDDS); Electronic Communication of Surveillance in Scotland (ECOSS); Electronic Health Record (HER); Electronic Prescribing Database (EPD); Emergency Care Data Set (ECDS); Emergency Department Data Daily (EDDD); Emergency Department Dataset (EDDS); General Practice (GP); Health and Social Care (HSC) Trusts; Hospital Electronic Prescribing and Medicines Administration (HEPMA); Hospital Episode Statistics (HES);  ICNARC – Intensive Care National Audit & Research Centre (All admissions) (ICNC); ICNARC – Intensive Care National Audit & Research Centre (COVID-19 Only admissions) (ICCD); Index of Multiple Deprivation (IMD); Intensive Care Admission (ICU); Intensive Care National Audit & Research Centre (ICNARC); Laboratory Information System (LIS); Maternal Indicators DataSet (MIDS); Maternity Services Dataset (MSDS); National Community Child Health database (NCCH); National Health Applications and Infrastructure Services (NHAIS); National Health Service (NHS); National Immunisation Management Service (NIMS); National Records of Scotland (NRS); Northern Ireland Maternity System (NIMATS); Northern Ireland Regional Accident and Emergency System (NIRAES); Office for National Statistics (ONS); Pathology data COVID-19 Daily (PATD); Patient Episode Database for Wales (PEDW); Prescribing Information System (PIS); Public Health England (PHE); Public Health Scotland (PHS); Royal College of General Practitioners Research and Surveillance Centre (RCGP RSC); Scottish Immunisation & Recall System (SIRS); Scottish Intensive Care Society Audit Group (SICSAG); Scottish Morbidity Record 01 (SMR01); Secondary Users Service (SUS); Turas Vaccination Management Tool (TVMT); Vaccine Management System (VMS); Welsh Demographic Service Dataset (WDSD); Welsh Longitudinal General Practice (WLGP) | | | | |

## Constructed variables

- Number of QCovid risk groups 0,1,2,3,4,5+
- Number of PCR tests in the pre vaccination era – 0,1,2,3-4, 5-9, 10+

## Consistency and error checking

We will check for implausible values for all variables used in the analyses, decide rules for cleaning them and document all data cleaning processes.

# Statistical analyses

The following sections are presented separately for each analytical objective.

## Objective a. Investigate the safety of Pfizer-BioNTech, Oxford-AstraZeneca and Moderna vaccines against mild-to-moderate and severe adverse events following immunisation, respectively.

### 5.1.1 Exposures of interest

First / second doses of Pfizer-BioNTech, Oxford-AstraZeneca or Moderna vaccines recorded in GP, NIMS, HSC Trusts, TVMT or CVVD (WIS) records.

### 5.1.2 Outcomes of interest

Mild-to-moderate and severe adverse events following immunisation (AEFI). Primary analysis will focus on GP consultations and hospitalisation due to any of the pre-specified adverse events of special interest. Other outcomes will include use of health services such as intensive care unit (ICU) admission, death, out-of-hours GP consultation, accident and emergency (A&E) department attendance. We will also include any immediate recording of side-effects in GP/TVMT records. Mild-to-moderate AEFI are likely to be systematically under-ascertained, especially those that are already known since patients are less likely to seek healthcare for well-known adverse events. Similarly, issues of increased reporting may occur in response to publicity around vaccine-related adverse events.

### 5.1.3 Potential confounders

The self-controlled case series study design addresses time invariant confounders. In case-control analyses, we will consider age, sex, region, socio-economic status (SES), residential settlement, comorbidities (e.g., asthma, chronic kidney disease, liver cirrhosis, chronic neurological condition, heart failure, diabetes (type 1 and type 2), dementia, coronary heart disease),[3] risk factors (smoking status, blood pressure, body mass index), and nursing home residential status.

5.1.4 Potential effect modifiers

We will consider time intervals of different lengths, sex, age bands, previous SARS-CoV-2 exposure and vaccine exposure categories as effect modifiers.

5.1.5 Analytical techniques

**5.1.5.1 Thromboembolic, haemorrhagic and idiopathic thrombocytopenic purpura (ITP) events**

Each UK nation will provide estimates from an incident-matched nested case-control study (NCCS). A generic inverse variance method will be used in the meta-analysis. We will use fixed-effect models since the same study designs will be carried out in each UK nation. Forest plots will be used to visualise the results. Statistical heterogeneity of our pooled vaccine safety estimates will be evaluated using the standard x^2^ and the I^2^ statistic.[4]

**5.1.5.2 Other adverse events**

For mild-to-moderate adverse events, the self-controlled case series (SCCS) study design will be used to determine the relative incidence of adverse events for exposed time periods (periods following vaccine administration) compared to unexposed time periods (pre- and post- vaccination periods unrelated to vaccination) in individuals who present with the outcome of interest (mild-to-moderate adverse events) (Appendix 1).[5]

Estimates for these events from each UK nation will also be pooled using the same analytical techniques for meta-analysis described in section 5.1.5.1.

### 5.1.6 Sub-group analysis

Subgroup analyses by vaccine type, age, sex, region, comorbidities, prior COVID-19 status, dose and dosing schedules will be considered.

### 5.1.7 Corrections for multiple testing

For analyses where we are testing a large number of hypotheses, the Benjamini-Hochberg procedure will be used to control the False Discovery rate.

### 5.1.8 Sensitivity analysis

In cases where there is media reporting on vaccine-related adverse events, we will consider a sensitivity analysis restricting the cohort end date to a time period prior to the reporting.

### 5.1.9 Other analysis

N/A

## Missing data

N/A

## Statistical software

All statistical tests are two sided and with a 5% significance level. All analyses will be carried out using R/Rstudio. The meta and metafor packages will be used for the analysis.

# Reporting results

## Reporting guidelines and conventions

Meta-analysis results will be reported according to the Strengthening the Reporting of Observational Studies in Epidemiology (STROBE) and Reporting of studies Conducted using Observational Routinely-collected Data (RECORD) guidelines. P-values will be quoted to two decimal places except if they are less than 0.001, in which case the p-value will be given as <0.001, or between <0.005 and >0.001, in which case they will be stated to three decimal places. Statistical estimates will be reported with 95% confidence intervals.

## Dissemination

The analysis will be written in a manuscript and submitted to a peer reviewed journal. We will also seek to provide near real-time reports on vaccine safety for the various vaccines to the funders and government COVID-19 advisory bodies as appropriate. All codes will be made publicly available via a GitHub repository.

# References

1. 73. Centers for Disease Control and Prevention. Vaccine safety. Rapid Cycle Analysis (RCA) to monitor the safety of COVID-19 vaccines in near real-time within the Vaccine Safety Datalink. <https://www.cdc.gov/vaccinesafety/pdf/VSD-1342-COVID19-RCA-Protocol_FinalV1.1_508.pdf>
2. Li X, Ostropolets A, Makadia R, et al. Characterizing the incidence of adverse events of special interest for COVID-19 vaccines across eight countries: a multinational network cohort study. BMJ. 2021;373:n1435.
3. Clift AK, Coupland CAC, Keogh RH, Diaz-Ordaz K, Williamson E, Harrison EM et al. Living risk prediction algorithm (QCOVID) for risk of hospital admission and mortality from coronavirus 19 in adults: national derivation and validation cohort study BMJ 2020; 371:m3731.
4. Higgins JP, Thompson SG, Deeks JJ, Altman DG. Measuring inconsistency in meta-analyses. BMJ. 2003;327(7414):557-560.
5. Farrington CP, Nash J, Miller E. Case series analysis of adverse reactions to vaccines: a comparative evaluation. *Am J Epidemiol* 1996; **143**(11): 1165-73.

# Appendix

**Appendix 1:** Schematic presentation of the self-controlled case series study design


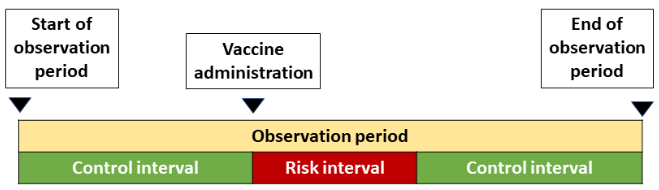

Supplement: S3 File — (DOCX) [file pmed.1003927.s003.docx]
