# Supplementary material for: First dose ChAdOx1 and BNT162b2 COVID-19 vaccinations and cerebral venous sinus thrombosis: A pooled self-controlled case series study of 11.6 million individuals in England, Scotland, and Wales
Source: PLoS Med. 2022 Feb 22;19(2):e1003927. doi: 10.1371/journal.pmed.1003927 (PMC8863261; doi:10.1371/journal.pmed.1003927)
Supplement: S4 File — Read Codes and SNOMED CT codes for CVST. CVST, cerebral venous sinus thrombosis. (DOCX) [file pmed.1003927.s004.docx]

**S4A Code lists. ICD-10 codes for CVST.**

| **Code** | **Description** |
| --- | --- |
| I636 | Cerebral infarction due to cerebral venous thrombosis, nonpyogenic |
| I676 | Nonpyogenic thrombosis of intracranial venous system |
| O225 | Cerebral venous thrombosis in pregnancy |
| O873 | Cerebral venous thrombosis in the puerperium |

**S4B Code lists. SNOMED CT codes for CVST.**

| **Code** | **Description** |
| --- | --- |
| 3681008 | Thrombophlebitis of torcular Herophili |
| 8166000 | Thrombophlebitis of basilar sinus |
| 15742000 | Thrombosis of inferior sagittal sinus |
| 18322005 | Thrombosis of torcular Herophili |
| 21258007 | Thrombosis of lateral venous sinus |
| 26954004 | Thrombophlebitis of superior sagittal sinus |
| 31314003 | Thrombophlebitis of cavernous venous sinus |
| 35386004 | Cavernous sinus syndrome |
| 42970005 | Nonpyogenic thrombosis of intracranial venous sinus |
| 48248005 | Thrombophlebitis of inferior sagittal sinus |
| 63795001 | Thrombosis of intracranial venous sinus of pregnancy AND/OR puerperium |
| 70607008 | Thrombosis of superior sagittal sinus |
| 84216001 | Cerebral venous thrombosis of pregnancy AND/OR puerperium |
| 88922007 | Thrombosis of basilar sinus |
| 89980009 | Thrombosis of cavernous venous sinus |
| 95455008 | Thrombosis of cerebral veins |
| 95461006 | Thrombophlebitis of cerebral vein |
| 106016005 | Intracranial sinus thrombosis, embolism AND/OR inflammation |
| 192759008 | Cerebral venous sinus thrombosis |
| 192760003 | Thrombosis of superior longitudinal sinus |
| 192761004 | Thrombosis transverse sinus |
| 192770001 | Thrombophlebitis of cavernous sinus |
| 192771002 | Thrombophlebitis of superior longitudinal venous sinus |
| 192772009 | Thrombophlebitis lateral venous sinus |
| 195229008 | Non-pyogenic venous sinus thrombosis |
| 200258006 | Obstetric cerebral venous thrombosis |
| 200259003 | Cerebral venous thrombosis in pregnancy |
| 200260008 | Cerebral venous thrombosis in the puerperium |
| 230222003 | Septic thrombophlebitis of straight sinus |
| 230223008 | Septic thrombophlebitis of sigmoid sinus |
| 230224002 | Septic thrombophlebitis of cortical vein |
| 230225001 | Septic thrombophlebitis of great cerebral vein |
| 230720005 | Cerebral venous thrombosis of straight sinus |
| 230721009 | Cerebral venous thrombosis of sigmoid sinus |
| 230722002 | Cerebral venous thrombosis of cortical vein |
| 230723007 | Cerebral venous thrombosis of great cerebral vein |
| 297157005 | Intracranial venous thrombosis |
| 302878004 | Intracranial septic thrombophlebitis |
| 302879007 | Septic thrombophlebitis of cavernous sinus |
| 302880005 | Septic thrombophlebitis of sagittal sinus |
| 302881009 | Septic thrombophlebitis of lateral sinus |
| 312586003 | Intracranial thrombophlebitis |
| 698627005 | Postoperative phlebitis and thrombophlebitis of intracranial sinuses |
| 702374000 | Neonatal noninfectious cerebral venous sinus thrombosis |
| 722930000 | Neonatal thrombosis of cerebral venous sinus |
| 871637001 | Thrombosis of multiple cerebral veins |
| 1078223005 | Thrombosis of cerebral venous sinus due to and following surgical procedure |
| 586551000000108 | Thrombosis of central nervous system venous sinus NOS |
| 586571000000104 | Thrombophlebitis of central nervous system venous sinus NOS |
| 756921000000102 | Cerebral vein thrombosis |

**S4C Code lists. Read codes for CVST.**

| **Code** | **Description** |
| --- | --- |
| G67A. | Cerebral vein thrombosis |
| F051z | Thrombosis of central nervous system venous sinus NOS |
| F053. | Thrombophlebitis of central nervous system venous sinuses |
| G676. | Nonpyogenic venous sinus thrombosis |
| F05 | Phlebitis and thrombophlebitis of intracranial sinuses |
| F050. | Embolism of central nervous system venous sinus |
| F0500 | Embolism cavernous sinus |
| F0501 | Embolism superior longitudinal sinus |
| F0502 | Embolism lateral sinus |
| F0503 | Embolism transverse sinus |
| F050z | Embolism central nervous system venous sinus NOS |
| F051. | Thrombosis of central nervous system venous sinuses |
| F0510 | Thrombosis cavernous sinus |
| F0511 | Thrombosis of superior longitudinal sinus |
| F0512 | Thrombosis lateral sinus |
| F0513 | Thrombosis transverse sinus |
| F052. | Phlebitis of central nervous system venous sinuses |
| F0520 | Phlebitis cavernous sinus |
| F0521 | Phlebitis of superior longitudinal sinus |
| F0522 | Phlebitis lateral sinus |
| F0523 | Phlebitis transverse sinus |
| F052z | Phlebitis of central nervous system venous sinus NOS |
| F0530 | Thrombophlebitis of cavernous sinus |
| F0531 | Thrombophlebitis of superior longitudinal venous sinus |
| F0532 | Thrombophlebitis lateral venous sinus |
| F053z | Thrombophlebitis of central nervous system venous sinus NOS |
| F05z. | Phlebitis or thrombophlebitis of CNS venous sinus NOS |
