# Supplementary material for: First dose ChAdOx1 and BNT162b2 COVID-19 vaccinations and cerebral venous sinus thrombosis: A pooled self-controlled case series study of 11.6 million individuals in England, Scotland, and Wales
Source: PLoS Med. 2022 Feb 22;19(2):e1003927. doi: 10.1371/journal.pmed.1003927 (PMC8863261; doi:10.1371/journal.pmed.1003927)
Supplement: S5 File — (DOCX) [file pmed.1003927.s005.docx]

**S4 Data pooling procedure.**

We detail the procedure that was used to pool the data using a fictional dataset for illustrative purposes.

Each country aggregated counts of incident CVST cases in the SCCS periods in the following manner

|  | England | Scotland | Wales |
| --- | --- | --- | --- |
| Reference | 1 | 1 | 0 |
| Pre-risk | 0 | 1 | 0 |
| Risk | 0 | 0 | 2 |

This information could be shared confidentially between trusted research environments (TREs) under the data governance agreements that were in place. These counts were then summed row-wise

|  | Total |
| --- | --- |
| Reference | 2 |
| Pre-risk | 1 |
| Risk | 2 |

This was expanded into a dataset with one row per individual and time period and synthetic IDs as follows

| Synthetic_ID | Period | Event | Period_length |
| --- | --- | --- | --- |
| 1 | Reference | 1 | 90 |
| 1 | Pre-risk | 0 | 14 |
| 1 | Risk | 0 | 28 |
| 2 | Reference | 1 | 90 |
| 2 | Pre-risk | 0 | 14 |
| 2 | Risk | 0 | 28 |
| 3 | Reference | 0 | 90 |
| 3 | Pre-risk | 1 | 14 |
| 3 | Risk | 0 | 28 |
| 4 | Reference | 0 | 90 |
| 4 | Pre-risk | 0 | 14 |
| 4 | Risk | 1 | 28 |
| 5 | Reference | 0 | 90 |
| 5 | Pre-risk | 0 | 14 |
| 5 | Risk | 1 | 28 |

Finally, the model was estimated using the clogit command from the survival package in R

clogit(Event ~ Period + offset(log(Period_length)) + strata(Synthetic_ID), data=data)
