# Supplementary material for: First dose ChAdOx1 and BNT162b2 COVID-19 vaccinations and cerebral venous sinus thrombosis: A pooled self-controlled case series study of 11.6 million individuals in England, Scotland, and Wales
Source: PLoS Med. 2022 Feb 22;19(2):e1003927. doi: 10.1371/journal.pmed.1003927 (PMC8863261; doi:10.1371/journal.pmed.1003927)
Supplement: S6 File — (DOCX) [file pmed.1003927.s006.docx]

**S6 Sensitivity analysis**

**Number of events and incidence rate ratios for CVST following first dose vaccination with ChAdOx1 and BNT162b2, excluding those who died within 90 days of their event**

| **Time period** | **Number of events** | **Incidence rate ratio (95% CI)** |
| --- | --- | --- |
| ChAdOx1 | | |
| Reference | 45 | 1 |
| Pre-risk | 9 | 1.29 (0.63-2.63) |
| Risk | 25 | 1.79 (1.10-2.91) |
| BNT162b2 | | |
| Reference | 29 | 1 |
| Pre-risk | <5 | 0.89 (0.31-2.52) |
| Risk | 7 | 0.78 (0.34-1.77) |

Event counts of <5 have been suppressed in accordance with disclosure control principles implemented by the data controllers. **CVST** – Cerebral venous sinus thrombosis; **CI** – Confidence interval. With the day of vaccination as day 0, the reference period was day -104 to day -14. The pre-risk period was day -14 to day 0. The risk period was day 0 to day 28.
