# Supplementary material for: First dose ChAdOx1 and BNT162b2 COVID-19 vaccinations and cerebral venous sinus thrombosis: A pooled self-controlled case series study of 11.6 million individuals in England, Scotland, and Wales
Source: PLoS Med. 2022 Feb 22;19(2):e1003927. doi: 10.1371/journal.pmed.1003927 (PMC8863261; doi:10.1371/journal.pmed.1003927)
Supplement: Attachment — Submitted filename: Response letter.docx [file pmed.1003927.s007.docx]

10 November 2021

Dear Louise,

**Re:** **PMEDICINE-D-21-03598R1**

Thank you for your welcome email dated 22 September 2021 inviting us to revise our paper. We are very grateful to your expert peer-reviewers and editors for their constructive feedback on our manuscript, which we have very carefully considered. We have made a number of revisions to the manuscript in the light of this feedback. For your convenience, we first reproduce the feedback received verbatim (in bold) before detailing our responses. Our changes/additions are in italics. We provide both a tracked version of the manuscript and a clean unmarked copy. Line numbers refer to the tracked version of the revised manuscript.

We provide an updated analysis with the cohort end dates extended to 30 June 2021.

**Requests from the editors:**

**General comments:**
**Throughout the paper, please adapt reference call-outs to the following style: "... ChAdOx1 vaccine, if available [11,12]." (noting the absence of spaces within the square brackets).**

**Response:** We have updated the citations to reflect this style.

**Title: Please revise your title according to PLOS Medicine's style. We suggest “First dose ChAdOx1 and BNT162b2 COVID-19 vaccinations and cerebral venous sinus thrombosis: A pooled self-controlled case series study of 12 million individuals in England, Scotland and Wales” or similar**

**Response:** We have revised the title of the paper to “First dose ChAdOx1 and BNT162b2 COVID-19 vaccinations and cerebral venous sinus thrombosis: A pooled self-controlled case series study of 11.6 million individuals in England, Scotland and Wales”. The count of people in the cohort has changed slightly due to the fact that in Scotland the number of people in GP records exceeds the entire population, due to people being present in GP records that are no longer resident in the country; we have now weighted the cohort using previously described methods to take account of this.[1]

**Abstract:**
**Abstract Background: Please specify which COVID-19 vaccines were restricted.**

**Response:** We have amended the abstract as follows (Lines 50-53)

*‘Several countries restricted administration of ChAdOx1 to older age groups in 2021 over safety concerns following case reports and observed vs. expected analyses suggesting a possible association with cerebral sinus venous thrombosis (CVST)’*

**“England, Scotland and Wales” would be preferable to “Great Britain”**

**Response:** As suggested, we have replaced “Great Britain” with “England, Scotland and Wales” throughout the manuscript.

**Abstract Methods and Findings:**
**Please provide brief demographic details of the study population (e.g. sex, age, ethnicity, etc)**
**Please provide the exact number of participants, population, the dates between which the study took place, length of follow up, and main outcome measures.**
**If possible, please provide the actual incidence of CVST in the study population in addition to IRRs**

**Line 62 - Please revise to “Vaccination with ChAdOx1 was associated with an elevated risk…”**

**Response:** We have re-written the ‘Methods and Findings’ section of the abstract to include the exact number of people in the cohort, the population they are drawn from, the cohort start and end dates, the total duration of follow-up and main outcome measure (Lines 59-63). We have also included new cohort descriptive tables for each nation, which contain demographic details of the study population, including incidence of CVST (Tables 1-3).

**In the last sentence of the Abstract Methods and Findings section, please describe 2-3 of the main limitations of the study's methodology.**

**Response:** We have added the following text to the ‘Methods and Findings’ section (Lines 79-86):

*‘The self-controlled case series study design implicitly controls for variables that are constant over the observation period, but also assumes that outcome events are independent of exposure. This assumption may not be satisfied in the case of CVST, firstly because it is a serious adverse event, and secondly because the vaccination programme in the UK prioritised the clinically extremely vulnerable and those with underlying health conditions, which may have caused a selection effect for individuals more prone to CVST. Although we pooled data from several large datasets, there was still a low number of events, which may have caused imprecision in our estimates.’*

**Abstract Conclusions:
Please begin your Abstract Conclusions with "In this study, we observed ..." or similar, to summarize the main findings from your study, without overstating your conclusions. Please emphasize what is new and address the specific implications of your study, being careful to avoid assertions of primacy and general statements such as "these results have implications for policy".**

**Response:** We have amended the Abstract Conclusions section as follows (Lines 89-93):

*‘In this study, we observed a small, elevated risk of CVST events following vaccination with ChAdOx1, but not BNT162b2. Our analysis pooled information from large datasets from England, Scotland and Wales. This evidence may be useful in risk-benefit analyses of vaccine policies, and to provide quantification of risks associated with vaccination to the general public.’*

**Author Summary:**
**At this stage, we ask that you include a short, non-technical Author Summary of your research to make findings accessible to a wide audience that includes both scientists and non-scientists. The Author Summary should immediately follow the Abstract in your revised manuscript. This text is subject to editorial change and should be distinct from the scientific abstract. Please see our author guidelines for more information: https://journals.plos.org/plosmedicine/s/revising-your-manuscript#loc-author-summary**
**In the final bullet point of ‘What Do These Findings Mean?’, please describe the main limitations of the study in non-technical language.**

**Response:** We have added the following Author Summary (Lines 96-117):

***‘Author Summary***

***Why Was This Study Done?***

- *There have been indications of a possible associations between COVID-19 vaccines – in particular, Oxford-AstraZeneca (ChAdOx1) – and cerebral venous sinus thrombosis, which is a rare type of blood clot in the brain.*
- *Further evidence on this topic is required in order to inform vaccine policy deliberations.*

***What Did The Researchers Do and Find?***

- *We estimated the rate of cerebral venous sinus thrombosis events in the four weeks following first dose vaccination compared with a 90-day period prior to vaccination.*
- *The rate of cerebral venous sinus thrombosis events in the four weeks following vaccination with Oxford-AstraZeneca was approximately twice as high compared to the baseline rate, implying an additional 0.25 events on average in this period per million people vaccinated.*
- *We did not find an association between Pfizer-BioNTech (BNT162b2) and cerebral venous sinus thrombosis.*

***What Do These Findings Mean?***

- *We found evidence of a slight increased risk of cerebral venous sinus thrombosis following first dose of the Oxford-AstraZeneca vaccine, but not the Pfizer-BioNTech vaccine.* *Public health authorities may wish to take this evidence into account when communicating the benefits and risks associated with COVID-19 vaccines to the general public.*

**Methods:
Please state that your study had a prospective statistical analysis plan early in the Methods section, and please include the relevant prospectively written document with your revised manuscript as a Supporting Information file to be published alongside your study, and cite it in the Methods section. A legend for this file should be included at the end of your manuscript.**

**Response:** We have added the following text to the start of the Methods section (Line 169):

*‘We followed a pre-specified statistical analysis plan (S2 Statistical analysis plan).’*

**Changes in the analysis-- including those made in response to peer review comments-- should be identified as such in the Methods section of the paper, with rationale. If a reported analysis was performed based on an interesting but unanticipated pattern in the data, please be clear that the analysis was data-driven.**

**Response:** We have added the following text to the Methods section (Lines 193-199):

*‘In accordance with our statistical analysis plan, we initially sought to carry out a meta-analysis of estimates from each country. However, there were too few events in each country for this to be feasible. As a result, we undertook a pooled analyses of aggregate count data from each country. We sought to carry out both a self-controlled case series study, and a case-control study using pooled data. However, the case-control study would have required sharing of individual-level information, which was not permitted under the data governance rules implemented by each country’s TREs. Therefore, we focused on the pooled SCCS study.’*

We also added the following text to the ‘Statistical analysis’ section (Line 248-251)

*‘**Following a suggestion from one of the reviewers, we undertook two post-hoc sensitivity analyses: the first excluded any cases who died within 90 days of their event in order to explore the SCCS assumption of event-dependent exposure; and the second focused only on CVST cases identified in secondary care records.’*

**Please add the following statement, or similar, to the Methods: "This study is reported as per the REporting of studies Conducted using Observational Routinely-collected Data (RECORD) guideline (S1 Checklist)."**

**Response:** We have amended the text under the Reporting subsection of the Methods section as follows (Lines 263-267):

*‘This study is reported in accordance with the REporting of studies Conducted using Observational Routinely-collected Data (RECORD) guidelines (S5 Checklist).’* **Line 153 - sentence appears incomplete**

**Response:** Apologies for this cut-off sentence. We have amended it as follows (Lines 217-221)

*‘The outcome of interest was incident cases of CVST in the observation period. SNOMED codes were used to identify CVST events recorded in primary care electronic health records in England and Read codes (Version 2) were used in Scotland and Wales (S3 Code lists). International Classification of Disease (ICD-10) codes were used to identify CVST events in hospital admission records (S3 Code lists).’*

**Results:**
**Please provide a table showing the baseline characteristics of the study population as Table 1.**

**Response:**

We have now added detailed cohort descriptive tables for each country (Tables 1-3).

**Please replace ~ with approximately**

**Response:** We have replaced ‘~’ with ‘approximately’ throughout the Results section.

**Line 184 - please revise to ‘incident cases of CVST’**

**Response:** We have amended this line as follows (Lines 274-276):

*‘Amongst the approximately 4.33 million people total vaccinated with ChAdOx1, there were 45 incident cases of CVST during the 90-day reference period and 27 incident cases of CVST during the 28-day post-vaccine risk period.’*

**Please report the dates between which the study took place**

**Response:** We have added the following text to the beginning of the Results section (Line 270):

*‘The cohorts started on 8 December 2020, and ended on 30 June 2021’*

**Discussion:**
**Please present and organize the Discussion as follows: a short, clear summary of the article's findings; what the study adds to existing research and where and why the results may differ from previous research; strengths and limitations of the study; implications and next steps for research, clinical practice, and/or public policy; one-paragraph conclusion.**

**Response:** We have reorganised the Discussion section using the suggested structure. In particular, we have added the following text (Lines 318-323):

*‘There have hitherto been few population-based studies estimating the association between COVID-19 vaccines and CVST. Such analyses are made challenging by the extreme rarity of CVST. Our study corroborates previous observed-expected analyses from the EMA [7], the Mayo Clinic Health System [8], a population-based study in Denmark and Norway [9], and a self-controlled case series using the QResearch database in England [10]. Our results are broadly consistent with the latter study, as would be expected due to the similar study design and population.’*

**Line 202 - please temper assertions of primacy by adding ‘to the best of our knowledge’ or similar. “England, Scotland and Wales” would be preferable to “Great Britain”**

**Response:** We have removed the word ‘novel’, and replaced ‘Great Britain’ with ‘England, Scotland and Wales’. It now reads (Lines 314-316)

*‘Our pooled SCCS analysis of national datasets from England, Scotland and Wales found an elevated risk of CVST in the four-week period following vaccination with ChAdOx1.’*

**Tables:**
**Please define all abbreviations used in the table legend of each table.**

**Response:** We have now added definitions for all abbreviations in the legend of each table.

**References:**
**Please ensure that journal name abbreviations match those found in the National Center for Biotechnology Information (NCBI) databases, and are appropriately formatted and capitalised.**
**Please also see https://journals.plos.org/plosmedicine/s/submission-guidelines#loc-references for further details on reference formatting, e.g. six authors are names prior to ‘et al’.**
**Where websites are cited, please specify the date of access**

**Response:** The references have been amended as suggested. **Supplementary files:
Please see** [**https://journals.plos.org/plosmedicine/s/supporting-information**](https://journals.plos.org/plosmedicine/s/supporting-information) **for our supporting information guidelines.

Response:** We have placed each item of supporting information in separate files and updated the formatting in accordance with the guidelines.

**Comments from the reviewers:**

**Reviewer #1: "First dose ChAdOx1 and BNT162b2 COVID-19 vaccinations and cerebral venous sinus thrombosis: pooled self-controlled case series of UK datasets" is a concise self-controlled case series (SCCS) analysis on incident cerebral venous sinus thrombosis (CVST) events, on over 12 million individuals from England, Scotland and Wales. The extremely low incidences for CVST (<50 for 4.33 million ChAdOx1 doses, <15 for 2.32 million BNT162b2 doses) has made any conclusive determination of increased risk challenging. With the SCCS design that implicitly controls for time-invariant variables (by effectively comparing every individual against himself before [including a clearance period] and after dosing), a 2.66 (1.48-4.79) incidence rate ratio (IRR) was found for ChAdOx1, against 1.07 (0.22-5.31) for BNT162b2 (Table 2).**

**While the study framework appears to allow for the analysis of other potential side effects in addition to CVST, this submission remains a timely addition to the literature, with further work on mRNA-1273 and further doses already proposed (Line 236).**

**Response:** Thank you for your kind comments.

**Still, a few issues might be considered:**

**1. While the description of the data compilation is relatively comprehensive (Figure 1), the detailed selection of the cohort might stand to be defined in greater detail. For example, the starting point (all individual given at least one dose of either the ChAdOx1 or BNT162b2 vaccine?) and any exclusions (e.g. missing data) might be presented in a flowchart.**

**Response:** We now include a flow chart indicating the data used in the analysis (Figure 2).

**2. While simple cohort demographics are provided in Table 1, it appears relevant to also present other demographic variables (e.g. age, gender, ethnicity, etc.) stratified by vaccine received, which would moreover help to give an idea of whether the demographics for each vaccine are comparable (all the more as their administration appears non-random from S1 Table).**

**Moreover, it appears possible that the underlying demographics may remain important in the analysis of IRR (related to the selection bias raised in Line 218), despite the SCCS study design. For example, assume that a rare disease has effectively zero incidence below a certain age (e.g. 50 years). Then, if Treatment A were provided mostly to those below that age, and Treatment B mostly to those above that age, then the potential for incidence would appear biased towards Treatment B (before considerations on whether increased disease incidence due to vaccine dosing is itself affected by age and other variables). Notably, from Table 2, given the 25 pre-risk events for ChAdOx1 (4.33 million doses), about 13 pre-risk events would be expected for BNT162b2 (2.32 million doses). However, only 6 pre-risk events were observed for BNT162b2 in reality. A fuller evaluation of the demographics for each vaccine group might therefore be warranted.**

**Response:** We now include Tables 1-3, with cohort characteristics for each country stratified by vaccine type.

**3. Data coverage would seem to remain somewhat of a concern, especially for the English data (~10% GP coverage, from Figure 1). In particular, the validity of SCCS would seem to assume individuals remaining "in system" throughout, i.e. if CVST were to occur for an individual, this CVST incidence would be expected to be recorded whether in the pre-risk, clearance or risk period. Would this be a reasonable expectation, or is it possible that CVST be treated at a venue that is not included in the databases used (e.g. the ~90% of GP practices not covered, for England)?**

**Response:** Thank you for raising this interesting point.It is indeed possible that individuals initially in the English RCGP data had CVST events recorded at GPs that are not in our coverage. However, the SCCS estimates are unchanged by addition of an arbitrary number of non-events to the dataset. Furthermore, it is also true that individuals initially outside the RCGP dataset may have had CVST events recorded in the RCGP data. If individuals move between general practices, their electronic health records are transferred to the new general practice, and therefore their date of event and date of vaccination will be present in the data. A sample selection bias may have occurred if CVST events recorded at GPs in our data were statistically distinct from CVST events recorded at GPs not in our data. However, the data from England are from a database of English GP practices that have been recruited to be nationally representative of the English population.[2,3] Therefore we do not believe this is a major concern.

**Reviewer #2: Thank you for the opportunity to review this manuscript on a matter of importance to public health.**
**Major comments:**
**1. 34 authors? Please elaborate.**

**Response:** All authors meet the ICJME authorship criteria in this unique collaborative effort between multiple data centres across the UK. This collaboration includes colleagues at several universities and public health authorities. Within each country, multiple people have been involved in setting up governance and secure infrastructure for hosting/accessing the datasets. Many analysts have been involved in cleaning a number of data streams that are combined to produce a final dataset in each country. We also have patient and public involvement representatives working on the project.

**2. Overall, the manuscript appears rushed and needs more attention. See e.g. line 153?**

**Response:** Apologies for this cut-off sentence. We have amended it as follows (Lines 217-226)

*‘The outcome of interest was incident cases of CVST in the observation period. SNOMED codes were used to identify CVST events recorded in primary care electronic health records in England and Read codes (Version 2) were used in Scotland and Wales (S3 Code lists**). International Classification of Cisease (ICD-10) codes were used to identify CVST events in hospital admission records (S3 Code lists).’*

We have also thoroughly reviewed the paper to ensure there are no other typographical errors.

**3. I do not understand the statistical approach. It is unclear what aggregation in each country and then expansion with so-called synthetic ID's is. If this means that you have first aggregated the true events and then tried to reconstruct synthetic events I am concerned. Please provide more detail and references that this is a valid approach. Alternatively, please estimate the association in each country and combine the three using e.g. inverse variance weighting.**

**Response:** We apologise that this was unclear. We now make clear that every event in the pooled dataset that was used in the analysis was a real event that occurred in the reference, pre-risk or risk periods. Synthetic identifiers (IDs) were generated for the sole purpose of distinguishing distinct cases in the count data from each other. These synthetic IDs were used to label strata, which is required in the model estimation. The dataset that was fed into the model estimation is identical to what would have been used if we started off with all data in one trusted research environment (TRE). We have added the following text to the ‘Statistical analysis’ section (Lines 228-241):

*‘The data governance procedure of the TREs in each country did not allow for individual level data to be shared. However, we obtained permissions to confidentially share counts of individuals stratified by categories, on the understanding that these would be combined in a pooled count. For a self-controlled case series study of the rate ratios of incident events in the observation time periods that does not include any covariates, the only information that is required to estimate the conditional Poisson model is event counts in the reference, pre-risk and risk periods. Analysts in each country had full access to their country’s data. Counts of incident cases in the reference, pre-risk and risk periods stratified by vaccine type received were collated in each nation. These were then gathered in the Scottish TRE and expanded into a full dataset with one row per individual and observation time period. Synthetic IDs were generated in order to distinguish distinct incident cases in the count data from each other and to label the strata in the model. The process of pooling the data is explained in more detail in the supporting information (S4 Data pooling procedure).’*

We have also included additional supporting information that explains the data pooling and estimation procedure in detail using a fictional dataset for illustrative purposes (S4 Data pooling procedure).

**4. Please provide sensitivity analyses supporting that the assumptions of the SCCS analysis is not violated.**

**Response:** We carried out a sensitivity analysis excluding anyone who died within 90 days of their event. We have added the following text to the Results section (Lines 301-305):

*‘We sought to carry out a post-hoc sensitivity analysis focusing on CVST events identified in secondary care records only – however, there were too few events. We carried out a post-hoc sensitivity analysis excluding anyone who died within 90 days of their CVST event. The only estimate that changed from our main analysis was that for the risk period following vaccination with ChAdOx1; the IRR was 1.79 (95% CI 1.10-2.91).’*

**5. Please provide a figure with two panels: each panel showing a histogram of the timing of event compared to date of vaccination with the two vaccines.**

**Response:** Unfortunately, we cannot provide this information under the statistical disclosure policies implemented by each country’s TRE. This is because there are many counts of 1, which are disclosive of individual level information. This issue is exacerbated by the fact that we also cannot share individual level information between TREs, which precludes pooling data on event timings and then plotting a histogram. Rather, histograms can be plotted within each TRE using only that country’s data, but they cannot be shared if they are statistically disclosive. In addition, we believe that sharing this information would potentially be in conflict with PLOS Medicine’s guidelines on sharing human research participant data (<https://journals.plos.org/plosmedicine/s/data-availability>).

Although we cannot share these histograms, we were able to plot and inspect them within each country’s TRE. We have added the following text to the Results section (Lines 307-311):

*‘Within each nation, we plotted histograms of event counts in the observation period in order to explore the suitability of the pre-risk period to account for event-dependent exposure to vaccination. There appeared to be a reduction in events in the two-week pre-risk period, but no obvious reduction outside of that time period. We are unable to share these histograms due to the statistical disclosure principles enacted by the TREs that hosted this analysis.’*

**6. You need to convince the reader that the many SNOMED and READ codes you have included accurately reflect CVST. Have these been validated?**

**Response:** We have added the following text to the Outcomes section (Lines 221-226):

*‘SNOMED and Read codes have hierarchies that enable thromboembolic events to be grouped by their location. These enable recording of the diagnosis of an event of interest – for example, a thromboembolic event could be recorded as intracranial, with further subdivisions into CVST, intracranial thrombophlebitis and thrombosis of cerebral veins, within the SNOMED hierarchy. The code lists used in this study were drawn up by the EAVE II clinical team and validated by experts in neurology and haematology.*

**7. Are you including GP information? Can you please provide a sensitivity analysis of only cases identified in a hospital setting.**

**Response:** We have now attempted to estimate the model using only those CVST cases identified in hospital records. However, the number of events was low, and we could not therefore estimate IRRs for all time periods. We have added the following text to the Results section (Lines 301-303):

*‘We sought to carry out a post-hoc sensitivity analysis focusing on CVST events identified in secondary care records only – however, there were too few events to permit useful estimation of IRRs.’*

**8. What are you using laboratory data for?**

**Response:** We sought to conduct a pooled case-control study as a secondary analysis, where number of COVID-19 tests and whether or not the individual had previously tested positive for COVID-19 were included as covariates. Unfortunately, the data governance rules implemented by the TREs meant that we could not pool the data required to carry out this analysis. We have added the following text to the Methods section explaining this (Lines 196-199)

*‘We sought to carry out both a self-controlled case series study, and a case-control study using pooled data. However, the case-control study would have required sharing of individual-level information, which was not permitted under the data governance rules implemented by each country’s TREs. Therefore, we focused on the pooled SCCS study.’*

**9. You need to discuss your results in more detail in comparison to other studies. You report a <1 CVST case per 1 mil vaccinated. This is much less than what others have reported.**

**Response:** We have added the following text to the Discussion section (Lines 319-326):

*‘Our study corroborates previous observed-expected analyses from the EMA [7], the Mayo Clinic Health System [8], a population-based study in Denmark and Norway [9], and a self-controlled case series using the QResearch database in England [10]. Our results are broadly consistent with the latter study [10], as would be expected due to the similar study design and population. Our estimated rate ratios are however notably smaller than risk ratios reported in [7-9]. This is likely due to the fact that we conducted a self-controlled case series that implicitly controls for variables that are constant over the observation period, as opposed to an observed-expected analysis.’*

**10. You conclude "Public health officials should take account of this evidence in designing policies on vaccination." What do you mean? Elaborate please?**

**Response:** We have replaced this sentence with the following (Lines 374-376):

*‘This evidence may be useful in risk-benefit evaluations for vaccine-related policies, and in providing quantification of risks associated with vaccination to the general public.’*

**Minor comments:**
**11. incidence rate ratio instead of incident rate ratio**

**Response:** Thank you for this correction. We have now replaced ‘incident rate ratio’ with ‘incidence rate ratio’ throughout the manuscript.

**12. I am confused by the Sup table 1 mentioning Scotland only?**

**Response:** The information in that table applies to the whole of the UK, not just Scotland. We have fixed the error.

**13. In SCCS terminology, a pre-risk period is your clearance period and the reference period is your pre-risk period.**

**Response:** We have renamed the SCCS periods accordingly throughout the manuscript.

**Reviewer #3: This is a straightforward analysis and methodological sound.**

**Response:** Thank you.

**However, I fail to see the novelty of the data as the association between CVT and vaccination has already been reported. Nonetheless, the study provides estimates for the British population.**

**Response:** The self-controlled case series design used implicitly controls for individual characteristics whereas most previous studies have undertaken observed-expected analyses which are at risk of residual confounding from time invariant covariates. We also believe that there is value in corroborating safety signals seen in previous studies, particularly in the context of vaccines that are being rolled out with unprecedented rapidity and scale.

**Some important data is missing and could add relevant information. Please provide data on mortality, demographic differences, or association with coagulation disorders. You might consider presenting you findings as a brief report or as a letter.**
**The figure legends are somewhat misplaced in the text.**

**Response:** We now provide cohort descriptions for each country in Tables 1-3, including demographic data, mortality and the incidence of events of interest including thrombosis or pulmonary embolus, stratified by type of first-dose vaccine.

The opportunity to revise our paper in the light of this thoughtful and constructive feedback has helped improve our work for which we are grateful. We trust that these revisions are to your satisfaction and that the manuscript is now suitable for publication. Please do not however hesitate to contact us if you require any further revisions or clarification.

With kind regards,

Aziz Sheikh and Steven Kerr, on behalf of the co-authors

**References**

1. Mulholland R H, Vasileiou E, Simpson CR, Robertson C, Ritchie L D, Agrawal U et al, Cohort Profile: Early Pandemic Evaluation and Enhanced Surveillance of COVID-19 (EAVE II) Database, *International Journal of Epidemiology*, Volume 50, Issue 4, August 2021, Pages 1064–1074, <https://doi.org/10.1093/ije/dyab028>
2. [de Lusignan](https://www.ncbi.nlm.nih.gov/pubmed/?term=de Lusignan S%5BAuthor%5D&cauthor=true&cauthor_uid=33605892) S, Bernal J L, Byf[ord](https://www.ncbi.nlm.nih.gov/pubmed/?term=Byford R%5BAuthor%5D&cauthor=true&cauthor_uid=33605892) R, [Amirthalingam](https://www.ncbi.nlm.nih.gov/pubmed/?term=Amirthalingam G%5BAuthor%5D&cauthor=true&cauthor_uid=33605892) G, [Filipa Ferreira](https://www.ncbi.nlm.nih.gov/pubmed/?term=Ferreira F%5BAuthor%5D&cauthor=true&cauthor_uid=33605892) F et al, Influenza and Respiratory Virus Surveillance, Vaccine Uptake, and Effectiveness at a Time of Cocirculating COVID-19: Protocol for the English Primary Care Sentinel System for 2020-2021, [JMIR Public Health Surveill.](https://www.ncbi.nlm.nih.gov/pmc/articles/PMC7899204/) 2021 Feb; 7(2): e24341. doi: 10.2196/24341.
3. Correa A, Hinton W, McGovern A, van Vlymen J, Yonova I, Jones S, de Lusignan S. Royal College of General Practitioners Research and Surveillance Centre (RCGP RSC) sentinel network: a cohort profile. BMJ Open. 2016 Apr 20;6(4):e011092. doi: 10.1136/bmjopen-2016-011092.
