# Supplementary material for: First dose ChAdOx1 and BNT162b2 COVID-19 vaccinations and cerebral venous sinus thrombosis: A pooled self-controlled case series study of 11.6 million individuals in England, Scotland, and Wales
Source: PLoS Med. 2022 Feb 22;19(2):e1003927. doi: 10.1371/journal.pmed.1003927 (PMC8863261; doi:10.1371/journal.pmed.1003927)
Supplement: Attachment — Submitted filename: Response letter 2.docx [file pmed.1003927.s008.docx]

2 December 2021

Dear Louise,

**Re:** **PMEDICINE-D-21-03598R2**

Thank you for your welcome email dated 1 December 2021 inviting us to submit a revised version of our paper. For your convenience, we first reproduce the feedback received verbatim (in bold) before detailing our responses.

**Requests from the editors:
It is of concern that the results of your analysis appear to have changed substantially during your revision (Table 4) - in particular, the differences in numbers of events. Please provide clarification as to how the revised results were generated, including changes in the data used and methodology. This may be subject to statistical re-review.

Response:** The number of events increased between the first and second versions that were submitted because the cohort end date was extended from 31 March 2021 to 30 June 2021. The methodology of the study was unchanged.


**Reviewer #1: Figure 2 defining the pre-risk, clearance and risk periods appears unchanged from the previous revision (R1). However Table 4 for reference/pre-risk/risk events/incidence rate ratios appears wholly different from Table 2 from the previous revision (R1). This might be clarified, since it appears the core of the analysis.

Response:** The cohort end date was extended from 31 March 2021 in R1, to 30 June 2021 in R2. This has led to an updated set of results, which we report in Table 4. The methodology of the study did not change.
 **Reviewer #2: The authors have satisfactorily addressed my comments. Thank you.

Response:** Thank you. **Reviewer #3: all well addressed**

**Response:** Thank you.

We trust that these responses are to your satisfaction and that the manuscript is now suitable for publication. Please do not however hesitate to contact us if you require any further revisions or clarification.

With kind regards,

Aziz Sheikh and Steven Kerr, on behalf of the co-authors
