# Supplementary material for: First dose ChAdOx1 and BNT162b2 COVID-19 vaccinations and cerebral venous sinus thrombosis: A pooled self-controlled case series study of 11.6 million individuals in England, Scotland, and Wales
Source: PLoS Med. 2022 Feb 22;19(2):e1003927. doi: 10.1371/journal.pmed.1003927 (PMC8863261; doi:10.1371/journal.pmed.1003927)
Supplement: Attachment — Submitted filename: Response letter 3.docx [file pmed.1003927.s009.docx]

15 January 2022

Dear Louise,

**Re:** **PMEDICINE-D-21-03598R2**

Thank you for your welcome email dated 14 January 2022 accepting our paper for publication conditional on fixing the remaining editorial and production issues. For your convenience, we first reproduce the feedback received verbatim (in bold) before detailing our responses. Paragraph numbers refer to the unmarked version.

**Requests from Editors:

Abstract Methods and Findings:
Please revise to “6,808,293 person years of follow-up”**

**Response:** We have made the requested change.

**If possible, please provide a brief demographic summary of the study population(s) (e.g. % female, mean age (SD), etc)**

**Response:** We are unable to do this as well as the other requested changes while staying within the 500 word limit for the abstract.

**Please specifically use the term ‘limitations’ in section beginning “The self-controlled case series study design…”**

**Response:** We have added the sentence directly preceding the indicated sentence (Page 4 Para 1):

*‘Our study had some limitations.’*

**Author Summary:
Please revise to “following first dose vaccination with either ChAdOx1 or Pfizer BioNTech (BNT162b2)”, or similar**

**Response:** We have amended the sentence to (Page 5 Para 6):

*‘…following first dose vaccination with ChAdOx1, but not BNT162b2.’*

**Please separate the text under ‘What Do These Findings Mean?’ into two separate bullet points.**

**Response:** We have made the requested change

**Please add a final bullet point to ‘What Do These Findings Mean?’ to describe the main limitations of the study in non-technical  language.**

**Response:** We have added the following bullet point (Page 6 Para 1):

*‘Our study had some limitations. Although the cohort was large, CVST is an extremely rare event, and this may have caused some imprecision in our estimates. In addition, there is a possibility that our model assumptions were not satisfied.’*

**Introduction:
Please revise to “...United States found a relative risk of 1.50 (95% CI 0.28-7.10) of CVST …”**

**Response:** We have made the requested change.

**Methods
Please provide your STROBE/RECORD checklist as (S1 Checklist)**

**Response:** We have renamed the Supporting information containing the checklist to ‘S1 Checklist’. **Results:
Please revise “4.95 million people total vaccinated”**

**Response:** We have removed the word ‘total’. The sentence now reads (Page 12 Para 3):

*‘Amongst the approximately 4.95 million people vaccinated with ChAdOx1…’*

**PLOS does not permit ‘data not shown’; please provide the results generated in your  sensitivity analysis (perhaps in a supplementary file)**

**Response:** We now provide a table with the results of our sensitivity analysis excluding those who died with 90 days of their event in new supporting information S6 Sensitivity analysis.  **Tables:
Please define all abbreviations used in the table legend of each table e.g. COPD, BMI, CVST and so on.

Response:** We now spell out all abbreviations used in the tables in their captions. **Table 1 - please clarify what is meant by ‘Age (years%)’; please clarify whether mean(SD) and Median(IQR) are presented and whether % signs are appropriate for Age and Average Household Age
Table 2 - please clarify what is meant by ‘Age (years%)’**

**Response:** The percentage sign following ‘years’ is a typo and has been removed. We now present only mean and standard deviation of age in order to be consistent in the three cohort descriptive tables.

**Table 4 - please provide a reminder in the table legend as to what the different time periods represent (i.e.  reference, pre-risk, risk)**

**Response:** We now include the following in text in the caption of Table 4 (Page 25):

*‘With the day of vaccination as day 0, the reference period was day -104 to day -14. The pre-risk period was day -14 to day 0. The risk period was day 0 to day 28.*’ **References:
Please try to be consistent in reference formatting - a DOI number for the full-text article is acceptable as an alternative to or in addition to traditional volume and page numbers.
Please remove italicised text e.g. ref 11 and report journal names as found at** [**http://www.ncbi.nlm.nih.gov/nlmcatalog/journals**](http://www.ncbi.nlm.nih.gov/nlmcatalog/journals) **e.g. Lancet (ref 15), N Engl J Med (refs 1, 2, etc)
Please see** [**https://journals.plos.org/plosmedicine/s/submission-guidelines#loc-references**](https://journals.plos.org/plosmedicine/s/submission-guidelines#loc-references) **for further details on reference formatting. 

Response:** We have updated the references to be in conformance with the provided guidance

**Supplementary files:
Please provide your STROBE/RECORD checklist as (S1 Checklist)**

**Response:** We have renamed the Supporting information containing the checklist to ‘S1 Checklist’.

We trust that these responses are to your satisfaction and that the manuscript is now suitable for publication. Please do not however hesitate to contact us if you require any further revisions or clarification.

With kind regards,

Aziz Sheikh and Steven Kerr, on behalf of the co-authors
